# Linked patterns of biological and environmental covariation with brain structure in adolescence: a population-based longitudinal study

**DOI:** 10.1038/s41380-020-0757-x

**Published:** 2020-05-22

**Authors:** Amirhossein Modabbernia, Abraham Reichenberg, Alex Ing, Dominik A. Moser, Gaelle E. Doucet, Eric Artiges, Tobias Banaschewski, Gareth J. Barker, Andreas Becker, Arun L. W. Bokde, Erin Burke Quinlan, Sylvane Desrivières, Herta Flor, Juliane H. Fröhner, Hugh Garavan, Penny Gowland, Antoine Grigis, Yvonne Grimmer, Andreas Heinz, Corinna Insensee, Bernd Ittermann, Jean-Luc Martinot, Marie-Laure Paillère Martinot, Sabina Millenet, Frauke Nees, Dimitri Papadopoulos Orfanos, Tomáš Paus, Jani Penttilä, Luise Poustka, Michael N. Smolka, Argyris Stringaris, Betteke M. van Noort, Henrik Walter, Robert Whelan, Gunter Schumann, Sophia Frangou, IMAGEN Consortium

**Affiliations:** 1 Department of Psychiatry, Icahn School of Medicine at Mount Sinai, New York, NY USA; 2 Department of Environmental Medicine & Public Health, Icahn School of Medicine at Mount Sinai, New York, NY USA; 3 Population Neuroscience and Precision Medicine, Institute of Psychiatry, Psychology and Neuroscience, King’s College London, London, UK; 4 Institute of Psychology, University of Bern, Bern, Switzerland; 5 Institut National de la Santé et de la Recherche Médicale, INSERM Unit 1000 “Neuroimaging & Psychiatry”, University Paris Saclay, University Paris Descartes - Sorbonne Paris Cité, Paris, France; 6 Psychiatry Department 91G16, Orsay Hospital, Orsay, France; 7 Department of Child and Adolescent Psychiatry and Psychotherapy, Central Institute of Mental Health, Medical Faculty Mannheim, Heidelberg University, Square J5, 68159 Mannheim, Germany; 8 Department of Neuroimaging, Institute of Psychiatry, Psychology & Neuroscience, King’s College London, London, UK; 9 Department of Child and Adolescent Psychiatry and Psychotherapy, University Medical Center, Göttingen, Germany; 10 Discipline of Psychiatry, School of Medicine and Trinity College Institute of Neuroscience, Trinity College Dublin, Dublin, Ireland; 11 Centre for Population Neuroscience and Precision Medicine (PONS), Institute of Psychiatry, Psychology & Neuroscience, SGDP Centre, King’s College London, London, UK; 12 Institute of Cognitive and Clinical Neuroscience, Central Institute of Mental Health, Medical Faculty Mannheim, Heidelberg University, Square J5, Mannheim, Germany; 13 Department of Psychology, School of Social Sciences, University of Mannheim, 68131 Mannheim, Germany; 14 Department of Psychiatry and Neuroimaging Center, Technische Universität Dresden, Dresden, Germany; 15 Departments of Psychiatry and Psychology, University of Vermont, Burlington, VT 05405 USA; 16 Sir Peter Mansfield Imaging Centre School of Physics and Astronomy, University of Nottingham, University Park, Nottingham, UK; 17 NeuroSpin, CEA, Université Paris-Saclay, 91191 Gif-sur-Yvette, France; 18 Department of Psychiatry and Psychotherapy CCM, Charité – Universitätsmedizin Berlin, corporate member of Freie Universität Berlin, Humboldt-Universität zu Berlin, and Berlin Institute of Health, Berlin, Germany; 19 Physikalisch-Technische Bundesanstalt (PTB), Braunschweig/Berlin, Germany; 20 Institut National de la Santé et de la Recherche INSERM Unit 1000 “Neuroimaging & Paris Saclay, University Paris Descartes and Maison de Solenn, Paris, France; 21 Institut National de la Santé et de la Recherche Médicale, INSERM Unit 1000 “Neuroimaging & Psychiatry”, University Paris Sacaly, University Paris Descartes, Paris, France; 22 AP-HP.Sorbonne Université, Department of Child and Adolescent Psychiatry, Pitié-Salpêtrière Hospital, Paris, France; 23 Bloorview Research Institute, Holland Bloorview Kids Rehabilitation Hospital and Departments of Psychology and Psychiatry, University of Toronto, Toronto, ON M6A 2E1 Canada; 24 Department of Social and Health Care, Psychosocial Services Adolescent Outpatient Clinic Kauppakatu 14, Lahti, Finland; 25 Department of Child and Adolescent Psychiatry and Psychotherapy, University Medical Centre Göttingen, von-Siebold-Str., 537075 Göttingen, Germany; 26 National Institute of Mental Health/NIH, 15K North Drive, Bethesda, MD 20892 USA; 27 MSB Medical School Berlin, Hochschule für Gesundheit und Medizin, Siemens Villa, Berlin, Germany; 28 School of Psychology and Global Brain Health Institute, Trinity College Dublin, Dublin, Ireland; 29 PONS Research Group, Department of Psychiatry and Psychotherapy, Campus Charite Mitte, Humboldt University, Berlin, Germany; 30 Leibniz Institute for Neurobiology, Magdeburg, Germany; 31 Institute for Science and Technology of Brain-Inspired Intelligence (ISTBI), Fudan University, Shanghai, P.R. China; 32 Djavad Mowafaghian Centre for Brain Health, University of British Columbia, Vancouver, BC Canada

**Keywords:** Neuroscience, Psychology

## Abstract

Adolescence is a period of major brain reorganization shaped by biologically timed and by environmental factors. We sought to discover linked patterns of covariation between brain structural development and a wide array of these factors by leveraging data from the IMAGEN study, a longitudinal population-based cohort of adolescents. Brain structural measures and a comprehensive array of non-imaging features (relating to demographic, anthropometric, and psychosocial characteristics) were available on 1476 IMAGEN participants aged 14 years and from a subsample reassessed at age 19 years (*n* = 714). We applied sparse canonical correlation analyses (sCCA) to the cross-sectional and longitudinal data to extract modes with maximum covariation between neuroimaging and non-imaging measures. Separate sCCAs for cortical thickness, cortical surface area and subcortical volumes confirmed that each imaging phenotype was correlated with non-imaging features (sCCA *r* range: 0.30–0.65, all *P*_FDR_ < 0.001). Total intracranial volume and global measures of cortical thickness and surface area had the highest canonical cross-loadings (|*ρ*| = 0.31−0.61). Age, physical growth and sex had the highest association with adolescent brain structure (|*ρ*| = 0.24−0.62); at baseline, further significant positive associations were noted for cognitive measures while negative associations were observed at both time points for prenatal parental smoking, life events, and negative affect and substance use in youth (|ρ| = 0.10−0.23). Sex, physical growth and age are the dominant influences on adolescent brain development. We highlight the persistent negative influences of prenatal parental smoking and youth substance use as they are modifiable and of relevance for public health initiatives.

## Introduction

Adolescence is a critical period for brain maturation leading to adult levels of emotional self-regulation and cognitive control [1–3]. At the same time, this period of brain reorganization is also associated with increased vulnerability to psychopathology [4–6]; the incidence of psychiatric disorders increases exponentially after the age of 10 years with 75% of cases being diagnosed by age 24 years [7, 8]. Factors that influence adolescent brain development are therefore critical in forming the foundation for both positive and negative adult functional outcomes [4–6].

A substantial body of literature has documented the typical brain structural changes observed during adolescence; cortical thickness shows a largely monotonic decrease [9, 10], cortical surface area expands and subcortical structures show individual variation in terms of expansion and contraction [11, 12]. These developmental trajectories are shaped by the dynamic interplay between biologically programmed functions (“nature”) and social and physical exposures (“nurture”). Age and biological sex are implicitly associated with biologically programmed functions as normal adolescent development follows predictable timelines and is sexually dimorphic [10, 13, 14]. Key social and physical exposures known to influence adolescent brain organization include perinatal events [15, 16], parental socioeconomic status [17, 18], parenting style [19] and social adversity [20]. Additionally associations with brain structure have been noted for personal characteristics such as cognitive abilities [21, 22], personality and behavioural traits [23–25].

Despite progress, the current literature is limited in several respects. Prior studies have typically examined either a single or very few of the non-imaging factors that can influence adolescent brain development; this narrow focus ignores the fact that many of these factors may be correlated. Notably, multivariate analyses in adults have identified a “positive-negative” axis of covariation between brain phenotypes and multiple individual attributes; those that are considered positive (e.g., higher cognitive abilities) generally show positive covariation with imaging phenotypes while the opposite is the case for attributes or indicators considered negative (e.g. substance use) [26–28]. Such multivariate analyses of developmental data require large longitudinal samples, which have typically not been available in studies in youth [24, 29–31]. Therefore, the appropriate modelling of the multiple factors associated with adolescent brain development remains a key unmet priority [32].

To address these challenges, the current study applied sparse canonical correlation analysis (sCCA) [33], a machine learning technique, to define associations between adolescent brain structural development with a broad array of factors indicating biological programming (age and sex), personal attributes, and social and environmental influences. We capitalized on the rich database of the IMAGEN Study (https://imagen-europe.com/), which provided high-quality brain structural imaging data collected from a population-derived cohort of more than 2000 youth. In addition, the dataset includes non-imaging variables that describe participants’ demographic, anthropometric, lifestyle, psychometric and behavioural features as well as their family function and social circumstances. IMAGEN participants underwent the same comprehensive evaluation twice, at age 14 years and at age 19 years thus enabling us to identify factors associated with brain structure at baseline and also with developmental brain changes over the inter-scan interval. We hypothesized that the patterns of covariation identified here would largely follow a “positive-negative” axis of covariation previously shown in studies of young adults [30−32], which have also emphasized that negative influences of social adversity and substance exposure amongst environmental factors. Our aim was to quantify, in the same integrative model, the contribution of biological programmed variables (i.e., age and sex) and variables relating to personal, social and environmental factors.

## Subjects and methods

### Participants

We used data from IMAGEN participants evaluated at age 14 years (baseline) and at age 19 years (developmental change) in eight sites in England, France, Germany and Ireland. At each evaluation, participants had a structural magnetic resonance imaging (MRI) scan and a comprehensive assessment of their individual, social and family characteristics. Following strict quality control procedures, outlined in Supplementary Fig. S1, we selected those participants for whom high-quality imaging data were available at baseline (baseline sample: *n* = 1476) and at both baseline and follow-up assessments (developmental change sample: *n* = 714). Written informed consent was obtained from all participants as well as from their legal guardians. The study was approved by all local ethics committees separately. Table 1 and Supplementary Table S1 summarize the characteristics of participants at baseline and in the developmental change sample.

**Table 1:** Non-imaging characteristics, the total analysis sample and the developmental change subsample at their baseline assessment.

Variable	Analysis sample (*N* = 1476)	Developmental change subsample (*N* = 714)
Youth demographic and anthropometric features		
Sex (female)	819 (55%)	445 (62%)
Age (years)	14.45 (0.40)	14.45 (0.41)
Height (cm)	167.37 (7.86)	167.32 (7.81)
Weight (kg)	58.23 (10.92)	57.58 (10.29)
Body mass index	20.72 (3.24)	20.5 (2.99)
Pubertal Development Scale	13.07 (2.22)	13.2 (2.2)
Youth perinatal events		
Birth weight (g)	3424 (563)	3419 (553)
Maternal smoking during pregnancy	178 (15%)	76 (12%)
Paternal smoking during pregnancy	250 (21%)	108 (18%)
Maternal alcohol use during pregnancy	269 (22%)	142 (23%)
Maternal medical illness during pregnancy	91 (7%)	51 (8%)
Pregnancy and/or birth complications	230 (19%)	117 (19%)
Breastfed	1032 (84%)	535 (86%)
Youth mental health		
Presence of psychiatric diagnosis	192 (13%)	79 (11%)
Youth cognitive ability		
General intelligence *g*-factor (*z* score)	0.02 (0.97)	0.17 (0.88)
ESPAD: Average grade		
• 1: C−	12 (1%)	2 (0.3%)
• 2: C	28 (2%)	10 (2%)
• 3: C+	40 (3%)	17 (3%)
• 4: B−	48 (4%)	18 (3%)
• 5: B	131 (10%)	59 (9%)
• 6: B+	408 (32%)	178 (29%)
• 7: A−	444 (35%)	244 (39%)
• 8: A	150 (12%)	92 (15%)
ESPAD: Truancy	4.13 (1.56)	3.99 (1.69)
Youth personality and temperament		
NEO: Neuroticism	1.88 (0.58)	1.91 (0.57)
NEO: Extroversion	2.45 (0.43)	2.43 (0.44)
NEO: Openness	2.2(0.47)	2.24 (0.49)
NEO: Agreeableness	2.33 (0.4)	2.38 (0.4)
NEO: Conscientiousness	2.3(0.55)	2.36 (0.56)
DAWBA Social Aptitude Scale	24.43 (5.79)	24.61 (5.6)
TCI: Novelty seeking	111.22 (10.42)	110.63 (10.47)
Youth substance risk and use		
SURPS: Anxiety sensitivity	2.25 (0.46)	2.26 (0.46)
SURPS: Hopelessness	1.87 (0.41)	1.87 (0.42)
SURPS: Impulsivity	2.43 (0.44)	2.39 (0.44)
SURPS: Sensation seeking	2.77 (0.55)	2.74 (0.54)
ESPAD: Frequency of lifetime smoking		
• 0: Never	875 (69%)	459 (74%)
• 1: 1–2 times	178 (14%)	86 (14%)
• 2: 3–5 times	52 (4%)	19 (3%)
• 3: 6–9 times	40 (3%)	15 (2%)
• 4: 10–19 times	38 (3%)	16 (3%)
• 5: 20–39 times	18 (1%)	10 (2%)
• 6: 40 or more times	60 (5%)	15 (2%)
ESPAD: Smoking in the preceding 30 days		
• 0: Not at all	1127 (89%)	571 (92%)
• 1: less than 1 cigarette per week	56 (4%)	26 (4%)
• 2: less than 1 cigarette per day	28 (2%)	9 (1%)
• 3: 1–5 cigarettes per day	32 (2%)	8 (1%)
• 4: 6–10 cigarettes per day	9 (1%)	3 (0.5%)
• 5: 11–20 cigarettes per day	6 (0.5%)	3 (0.5%)
• 6: more than 20 cigarettes per day	3 (0.2%)	0 (0%)
ESPAD: Frequency of lifetime alcohol use		
• 0: Never	285 (23%)	135 (22%)
• 1: 1–2 times	324 (26%)	161 (26%)
• 2: 3–5 times	239 (19%)	127 (21%)
• 3: 6–9 times	173 (14%)	86 (14%)
• 4: 10–19 times	132 (10%)	68 (11%)
• 5: 20–39 times	64 (5%)	24 (4%)
• 6: 40 or more times	41 (3%)	17 (3%)
ESPAD: Frequency of alcohol use in the preceding 30 days		
• 0: Not at all	638 (51%)	325 (53%)
• 1–2 times	452 (36%)	218 (35%)
• 3–5 times	104 (8%)	46 (7%)
• 6–9 times	36 (3%)	16 (3%)
• 10–19 times	19 (1%)	10 (2%)
• 20–39 times	6 (0.5%)	2 (0.3%)
• 40 or more times	3 (0.2%)	1 (0.2%)
ESPAD: Frequency of lifetime cannabis use		
• 0: Never	1180 (94%)	592 (96%)
• 1: 1–2 times	41 (3%)	16 (3%)
• 2: 3–5 times	9 (1%)	3 (0.5%)
• 3: 6–9 times	8 (1%)	2 (0.3%)
• 4: 10–19 times	5 (0.3%)	0 (0%)
• 5: 20–39 times	1 (0.1%)	0 (0%)
• 6: 40 or more times	10 (1%)	3 (0.5%)
ESPAD: Frequency of cannabis use in the preceding 30 days		
• 0: 0	1211 (96%)	603 (98%)
• 1: 1–2 times	30 (2%)	11 (2%)
• 2: 3–5 times	2 (0.2%)	0 (0%)
• 3: 6–9 times	4 (0.3%)	0 (0%)
• 4: 10–19 times	3 (0.2%)	0 (0%)
• 5: 20–39 times	1 (0.1%)	0 (0%)
• 6: 40 or more times	3 (0.2%)	2 (0.3%)
Youth social and family circumstances		
LEQ: Total number of negative life events since last visit	5.83 (2.96)	5.66 (3.03)
LEQ: Family-related life events since last visit	0.26 (0.23)	0.24 (0.22)
LEQ: Family accidents or illness since last visit	0.53 (0.27)	0.51 (0.27)
LEQ: Events relating to sexuality/intimacy since last visit	0.29 (0.18)	0.28 (0.18)
LEQ: Autonomy: events relating to independence since last visit	0.53 (0.18)	0.53 (0.17)
LEQ: Deviance: events relating to legal or school problems	0.27 (0.23)	0.24 (0.23)
LEQ: Relocation: events relating to change in schools or residence since last visit	0.45 (0.33)	0.45 (0.33)
LEQ: Distress: distressing events since last visit	0.29 (0.19)	0.28 (0.19)
LEQ: Other events since last visit	0.33 (0.27)	0.34 (0.28)
ESPAD: Victim of bullying	0.19 (0.39)	0.2 (0.4)
ESPAD: Perpetrator of bullying	0.1 (0.3)	0.07 (0.26)
DAWBA: Family stressors: Financial/housing	0.72 (1.08)	0.65 (1.03)
DAWBA: Family stressors: Work pressure	1.07 (1.08)	1.11 (1.09)
DAWBA: Family stressors: Illness	0.52 (0.9)	0.51 (0.9)
DAWBA: Family stressors: Relationships/addiction	0.42 (0.74)	0.39 (0.73)
DAWBA: Child experience: Affirmation	10.82 (1.47)	10.9 (1.35)
DAWBA: Child experience: Discipline	3.43 (1.58)	3.35 (1.58)
DAWBA: Child experience: Rules	4.64 (1.24)	4.64 (1.27)
DAWBA: Living with both parents	1267 (86%)	620 (87%)
FIGS: Positive family history of psychiatric disorders	274 (19%)	129 (18%)
Parental characteristics		
ESPAD: Maternal education level		
• GCSEs or CSEs or below	235 (17%)	90 (13%)
• NVQ or GNVQ	273 (19%)	112 (16%)
• A levels or a BTEC national diploma	204 (15%)	111 (16%)
• Advanced diploma	203 (14%)	100 (15%)
• Bachelor degree	321 (23%)	175 (26%)
• Professional qualification (Master’s degree and above)	162 (11%)	91 (13%)
ESPAD: Paternal education level		
• GCSEs or CSEs or below	300 (21%)	113 (17%)
• GNVQ or NVQ	209 (15%)	109 (16%)
• A levels or a BTEC national diploma	175 (12%)	86 (13%)
• Advanced diploma	164 (12%)	78 (12%)
• Bachelor degree	313 (22%)	168 (25%)
• Professional qualification (Master’s degree and above)	237 (17%)	125 (18%)

Continuous variables are shown as mean (standard deviation); categorical variables are shown as number (percentage, %). Details of each variable are shown in Supplementary Table S2.

*ESPAD* European School Survey Project on Alcohol and Other Drugs, *DAWBA* Development and Well-being Assessment, *FIGS* Family Interview for Genetic Studies, *LEQ* Life Events Questionnaire, *NEO* NEO-Five Factor Personality Inventory, *SURPS* Substance Use Risk Profile Scale, *TCI* Temperament and Character Inventory, *WISC-IV* Wechsler Intelligence Scale for Children-IV, *GCSE* General Certificate of Secondary Education, *CSE* Certificate of Secondary Education, *GNVC* General National Vocational Qualification, *NVQ* National Vocational Qualification, *BTEC* Business and Technology Education Council, *A levels* Advanced Level Qualification.

### Non-imaging variables

We considered variables corresponding to youths’ demographic characteristics (age and sex), anthropomorphic features (height, weight, body mass index and pubertal stage), perinatal events (parental smoking/substance use, maternal medical conditions, birth complications, breastfeeding), mental health (presence or absence of psychiatric diagnoses), cognitive ability (general intelligence and scholastic performance), personality and temperament, substance use and risk, social and family circumstances (life events, bullying, family function, socioeconomic status, housing) and parental education level. Because our study focuses on general neurodevelopment, we did not include measures of individual psychopathologies (interested reader is referred to the paper by Ing et al. on that topic [34], a full list of IMAGEN publications is provided in Supplementary Material). Definitions of the variables and description of the assessment instruments are presented in Supplementary Table S2. Missing values for non-imaging features were imputed using random forest in R using available values from other non-imaging features (package missForest version 1.4) although the percentage of missing values was generally low (Supplementary Table S3).

### Neuroimaging acquisition and processing

High-resolution T_1_-weighted images were obtained at eight European sites (Berlin, Dresden, Dublin, Hamburg, London, Mannheim, Nottingham and Paris) with 3T MRI systems by different manufacturers (Siemens: four sites, Philips: two sites, General Electric: one site, and Bruker: one site). The MR protocols, cross-site standardization and quality control procedures of the IMAGEN study are described in Supplementary Material and in Schumann et al. [35]. In addition to the standard IMAGEN procedures, we also applied a validated automatic quality control algorithm (Qoala-T; https://github.com/Qoala-T/QC) [36] to preprocessed MRI scans to exclude low-quality images at each assessment wave (Supplementary Fig. S1). Subsequently, we used an automatic robust longitudinal processing pipeline [37] to extract reliable estimates of cortical thickness and surface area and subcortical volumes (Supplementary Table S4) using Freesurfer version 6.0 (https://surfer.nmr.mgh.harvard.edu/). The final baseline (*n* = 1476) and developmental change (*n* = 714) samples were defined following outlier exclusion undertaken in each sample separately using the Mahalanobis distance with a quantile cut-off of 0.999 implemented in chemometrics package, version 1.4.2, in R. Prior to statistical analysis, the imaging variables were adjusted for site/scanner effects using ComBat in R (https://github.com/Jfortin1/ComBatHarmonization) [38]. Initially used for batch adjustment of genetic data, ComBat uses Empirical Bayes to adjust for between-site variability while preserving biological variability.

### Statistical analysis

#### Descriptive statistics

For all variables, differences in baseline and follow-up values were examined using paired *t* tests or McNemar’s tests for continuous and categorical variables respectively.

#### Datasets

The neuroimaging and non-imaging datasets and their constituent variables were described above and in Supplementary Tables S2 and S4. Cortical thickness, cortical surface area, and subcortical volumes were examined separately because these phenotypes are genetically independent and follow different developmental trajectories [39–41]. Analyses of the baseline sample (*n* = 1476) included global neuroimaging measures (e.g. mean cortical thickness, total surface area, and total intracranial volume). Values of brain regional measures for each imaging phenotype (cortical thickness, area and subcortical volume) were thus not adjusted by their respective global measures. In the developmental change subsample (*n* = 714), we were interested in modelling the effect on the variables that changed between the baseline and follow-up assessments. In each IMAGEN participant, developmental change in any variable was calculated as: (follow-up value – baseline value), which was then residualized by the baseline value. Parental education and perinatal events were not included in the developmental change analyses as their values did not change between baseline and follow-up. Pubertal development and general intelligence (*g*-factor) were not included in the main developmental change analyses; these were not assessed at follow-up. We also performed additional sCCA on the developmental change data with these variables included. Sex was retained in the main model as it exerts a continuous influence on brain structural development during adolescence.

#### Identification of multivariate associations between imaging and non-imaging datasets

We used sparse canonical correlations analyses [33], which is a version of the general canonical correlation analysis (CCA), to identify linked dimensions between imaging and the non-imaging datasets (additional details in Supplementary Material). CCA is a method for finding relationships between two multivariate sets of variables, all measured in the same individuals [33]. CCA seeks to find linear combinations of variables from each dataset that are maximally correlated with each other (referred to as pairs of canonical variates or modes). Traditional CCA models are prone to overfitting and are not fully equipped to deal with variables that are correlated. Regularization is commonly employed to penalize the complexity of a learning model and control overfitting. sCCA implements regularization by using a sparsity parameter that penalizes some variables by setting their contribution to the overall model to zero. In addition to the pairs of variates (i.e. one variate from each dataset), sCCA generates information for variables with non-zero contributions. These are expressed as weights (i.e. magnitude of the contribution of the variable to the variate from the same dataset) and as canonical cross-loadings (i.e. coefficient of the correlation of the variable with the variate of the opposite dataset).

In this study, sCCA models were implemented in R version 6.8.0 using the *sgcca.wrapper* function from the mixOmics package. Non-imaging and neuroimaging variables were standardized to a mean of 0 and a standard deviation of 1 before being entered into the sCCA models [33, 42]. We then followed standard procedures to identify the optimal sparsity parameters for each sCCA model. For each analysis, we computed the sparse parameters by running the sCCA with a range of candidate values (from 1/√*p* to 1, at 10 increments, where *p* is the number of features in that view of the data) for each imaging and non-imaging dataset, and then fitted the resulting models. We selected the optimal sparse criteria combination based on the parameters that corresponded to the values of the model that maximized the sCCA correlation value. We then computed the optimal sCCA model and determined its significance based on exact *P* values calculated from 1000 random permutations. The *P* value was defined as the number of permutations that resulted in an equal or higher correlation than the original data divided by the total number of permutations (further details in Supplementary Material). Because we implemented multiple sCCA models throughout the manuscript, significance of each mode was further adjusted using false discovery correction (FDR). In addition, statistically significant modes were tested for reliability and reproducibility (described below) and only models that survived these analyses are reported. For significant sCCA mode, we report weights and loadings of the contributing variables if these are at least of small effect (>|0.1|) according to current standards [43].

#### Reliability, reproducibility and supplemental analyses

We undertook the following analyses to determine the robustness of our results: (i) we tested the association between image quality and canonical correlation coefficients. A quality score for each individual scan was calculated using the Qoala algorithm. We then computed the Spearman’s correlation coefficient between the mean data quality score and the sCCA -coefficients derived from 500 randomly resampled subsets of the original sample; (ii) we assessed the stability of the findings of each sCCA in relation to sample size and composition. To do so we repeated each sCCA in 100 randomly generated subsets each containing 10−150% of the original data in 10% increments (1500 subsamples in total); (iii) following our prior work [28], we calculated redundancy reliability (RR) scores for each sCCA; to achieve this we repeated each sCCA in 500 randomly generated subsets and quantified the reliability of canonical cross-loadings (details in Supplementary Material); (iv) we randomly sampled 50% of the data 500 times (training set), calculated sCCA on each training set and then used the weights from the sCCA on the training set on the remainder 50% of the data (test set) to calculate the canonical correlations in the test set. We reported only those modes that met the following robustness criteria: (i) statistically significant at an FDR-corrected *P* value < 0.001; (ii) had a median RR-score > 0.80, and (iii) average canonical correlation on the resampled test sets was at least 80% of that of the training sets. We performed additional sCCAs to evaluate the effect of removing sex and age on the results.

## Results

The non-imaging characteristics of the baseline sample and developmental change subsample are shown in Table 1 and Supplementary Table S1. The corresponding descriptive statistics for cortical thickness and area and subcortical volumes are presented in Supplementary Table S5. At a nominal statistical level, the follow-up subsample included more women (*P* < 0.001) and more offspring of parents with higher levels of parental education (*P* < 0.05) than the baseline sample (Table 1). Over the 5-year mean inter-scan interval, the mean (standard deviation, SD) of the global cortical thickness decreased by 0.12 (0.06) mm on the right and 0.13 (0.06) mm on the left. Total cortical surface area showed an average decrease of 3891(2274) mm^2^ during the same period.

### Linked imaging and non-imaging dimensions

#### Cortical thickness

##### Baseline

**Fig. 1:**
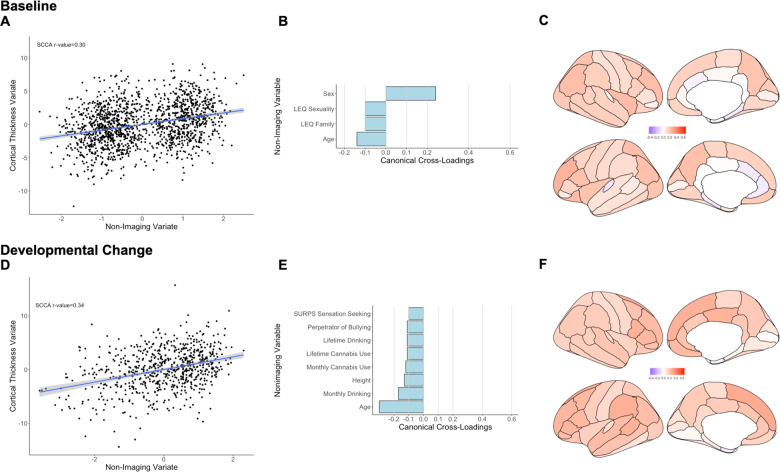
Sparse canonical correlation analysis (sCCA) for baseline and developmental change in cortical thickness. Upper panel: Baseline: **a** First canonical correlation coefficient. **b** Canonical cross-loadings for non-imaging variables. **c** Canonical cross-loadings for imaging variables. Lower panel: Developmental change: **d** First canonical correlation coefficient. **e** Canonical cross-loadings for non-imaging variables. **f** Canonical cross-loadings for imaging variables. LEQ Life Event Questionnaire, SURPS Substance Use Risk Profile Scale.

The sCCA testing the association between cortical thickness measures and non-imaging variables was significant (*r* = 0.30, *P*_FDR_ < 0.001, mean (SD) permuted *r* = 0.12(0.01)) (Fig. 1a) and accounted for 9% of the covariance (Supplementary Fig. S2). The canonical weights and cross-loadings for the imaging and non-imaging variables are shown in Supplementary Tables S6−S9. Sex and age had the highest positive canonical cross-loadings on the imaging variate while the frequency of negative family life events had the highest negative canonical cross-loading (Fig. 1b; Supplementary Table S7). Canonical cross-loadings of *ρ* > 0.10 were noted for nearly all cortical regions and were highest for the mean total cortical thickness and for the rostral middle frontal cortex (Fig. 1c; Supplementary Table S9).

##### Developmental change

The sCCA testing the association between developmental changes in cortical thickness measures and inter-scan changes in non-imaging variables was significant (*r* = 0.34, *P*_FDR_ < 0.001, mean (SD) permuted *r* = 0.16(0.02)) (Fig. 1d) and accounted for 12% of the covariance (Supplementary Fig. S3). The canonical weights and cross-loadings for the imaging and non-imaging variables are shown in Supplementary Tables S10−S13. Inter-scan changes in age, height, and frequency of alcohol and cannabis use had the highest negative canonical cross-loadings (Fig. 1e; Supplementary Table S11). Developmental changes in cortical thickness with canonical cross-loadings of *ρ* > 0.1 were noted in most cortical regions; the highest loadings were found in the superior frontal, the pars opercularis, supramarginal, bank of the superior temporal sulcus, and posterior cingulate cortices (Fig. 1f; Supplementary Table S13).

#### Cortical surface area

##### Baseline

**Fig. 2:**
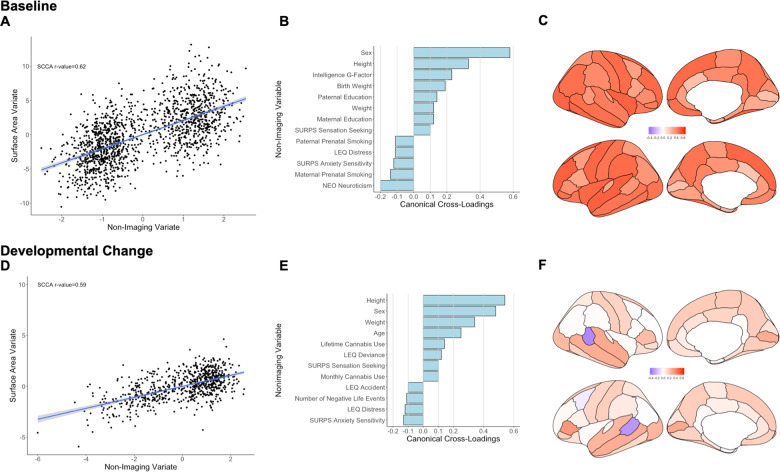
Sparse canonical correlation analysis (sCCA) for baseline and developmental change in cortical surface area. Upper panel: Baseline: **a** First canonical correlation coefficient. **b** Canonical cross-loadings for non-imaging variables. **c** Canonical cross-loadings for imaging variables. Lower panel: Developmental change: **d** First canonical correlation coefficient. **e** Canonical cross-loadings for non-imaging variables. **f** Canonical cross-loadings for imaging variables. LEQ Life Event Questionnaire, SURPS Substance Use Risk Profile Scale, NEO NEO-Five Factor Personality Inventory.

The sCCA testing for the association between cortical surface area measures and non-imaging variables was significant (*r* = 0.62, *P*_FDR_ < 0.001, mean (SD) permuted *r* = 0.12(0.01)) (Fig. 2a) and accounted for 38% of the covariance (Supplementary Fig. S4). The canonical weights and cross-loadings for the imaging and non-imaging variables are shown in Supplementary Tables S14−S17. The highest positive canonical cross-loadings were observed for sex, anthropometric measures (height, weight and birth weight), youth cognitive ability and parental education. The highest negative canonical cross-loadings were observed for youth neuroticism and anxiety sensitivity and parental perinatal smoking (Fig. 2b; Supplementary Table S15). Canonical cross-loadings with the non-imaging variate with *ρ* values ranging from 0.20 to 0.60 were noted for all cortical regions with the top five seen for the total surface area, and the surface area of the left superior temporal cortex, the left rostral middle frontal cortex, the right fusiform and the right insula (*ρ* = 0.50–0.60) (Fig. 2c; Supplementary Table S17).

##### Developmental change

The sCCA testing the association between developmental changes in cortical surface area and inter-scan changes in non-imaging variables was significant (*r* = 0.59, *P*_FDR_ < 0.001, mean (SD) permuted *r* = 0.20(0.02)) (Fig. 2d) and accounted for 35% of the covariance (Supplementary Fig. S5). The canonical weights and cross-loadings for the imaging and non-imaging variables are shown in Supplementary Tables S18−S21. Male sex, inter-scan changes in age and in anthropometric features (height and weight), cannabis use, and sensation seeking/deviance had the highest positive canonical cross-loadings with the imaging variate whereas anxiety sensitivity, distressing and negative life events had the highest negative canonical cross-loadings (Fig. 2e; Supplementary Table S19). Developmental changes in the surface area showed mostly positive and small to moderate canonical cross-loadings (*ρ* < 0.35) throughout the cortex; notable negative canonical cross-loadings were observed within the bank of the superior temporal gyrus bilaterally (Fig. 2f; Supplementary Table S21).

#### Subcortical volumes

##### Baseline

**Fig. 3:**
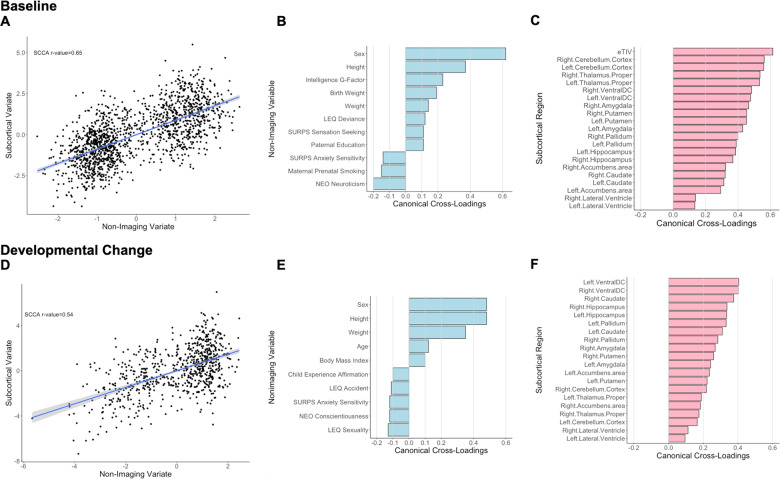
Sparse canonical correlation analysis (sCCA) for baseline and developmental change in subcortical volumes. Upper panel: Baseline: **a** First canonical correlation coefficient. **b** Canonical cross-loadings for non-imaging variables. **c** Canonical cross-loadings for imaging variables. Lower panel: Developmental change: **d** First canonical correlation coefficient. **e** Canonical cross-loadings for non-imaging variables. **f** Canonical cross-loadings for imaging variables. LEQ Life Event Questionnaire, SURPS Substance Use Risk Profile Scale, NEO NEO-Five Factor Personality Inventory.

The sCCA testing for the association between subcortical volumes and non-imaging variables was significant (*r* = 0.65, *P*_FDR_ < 0.001, mean (SD) permuted *r* = 0.12(0.01)) (Fig. 3a) and accounted for 42% of the covariance (Supplementary Fig. S6). The canonical weights and cross-loadings for the imaging and non-imaging variables are shown in Supplementary Tables S22−S25. Sex, youth’s general cognitive ability and anthropometric measures (height, birth weight, and weight) showed the highest positive canonical cross-loadings while maternal prenatal smoking and youth personality traits relating to anxiety and neuroticism showed the highest negative canonical cross-loading (Fig. 3b; Supplementary Table S23). Canonical cross-loadings with the non-imaging variate with *ρ* values ranging from 0.14 to 0.61 were noted for all subcortical regions with the top five being the total intracranial volume, the cerebellum and the thalami (Fig. 3c; Supplementary Table S25).

##### Developmental change

The sCCA testing the association between developmental changes in regional subcortical volumes was significant (*r* = 0.54, *P*_FDR_ < 0.001, mean (SD) permuted *r* = 0.18(0.02)) (Fig. 3d) and accounted for 29% of the covariance (Supplementary Fig. S7). The canonical weights and cross-loadings for the imaging and non-imaging variables are shown in Supplementary Tables S26−S29. Male sex, inter-scan changes in age and anthropometric measures (height, weight and body mass index) showed the highest positive canonical cross-loadings while life experiences related to sexuality and youth personality traits relating to anxiety and conscientiousness showed the highest negative canonical cross-loadings (Fig. 3e; Supplementary Table S27). Developmental changes in regional subcortical volumes with showed positive canonical cross-loadings with ρ values ranging from 0.10 to 0.40, with lateral ventricles having the smallest canonical cross-loadings (*ρ* range 0.10–0.12) (Fig. 3f; Supplementary Table S29).

### Reliability analysis

Only the first mode for each sCCA analysis passed the criteria for reporting (Supplementary Figs. S2−S7). Resampling analyses showed that the canonical correlations were largely stable for samples larger than 50% of the originals. The results of the stability and reliability analyses are summarized in Supplementary Figs. S8 and S9 and Supplementary Table S30. To quantify the associations beyond the effect of age and sex, we also reran the sCCA after regressing out age and sex from both imaging and non-imaging variables. We found that in most cases (except for cortical thickness at baseline), first canonical mode remained significant (Supplementary Table S31). Further sCCA analysis showed that among variables that were only measured at baseline, maternal education, pubertal stage, and history of being breastfed had significant association with the imaging variate (Supplementary Table S32).

## Discussion

We leveraged the rich dataset and a longitudinal design of the IMAGEN study to identify patterns of covariation between adolescent brain structure and youth personal attributes, lifestyle and psychosocial environment. Using integrated multivariate analyses, we demonstrate that adolescent brain structural development was most strongly associated with sex, age and anthropometric features. Contributions from environmental sources were quantitatively smaller and highlighted the influence of parental smoking and education, unpleasant life events and youths’ cognitive ability, use of alcohol and cannabis and personality traits related to negative affect.

We found that measures of cortical thickness, surface area and subcortical volumes show mostly unitary patterns of covariation that reflect the corresponding global measures. Regional and global brain structural measures, both at baseline and at follow-up, showed the highest covariation with sex, age and anthropometric measures. These associations have been consistently noted in prior research [14, 28, 44]. However, the integrated analyses implemented here enable the study of these factors in the wider context of other potential influences relating to environmental exposures. Thus, a novel finding of the current study is that biologically programmed processes relating to sexual dimorphism and the time-dependent evolution of development remain the most significant drivers of adolescent brain development even when accounting for other influences.

Beyond sex and age, our findings support previous reports in young adults, which have found that the pattern of covariation of brain-derived phenotypes largely recapitulates conventional notions of “positive” and “negative” influences [30, 31]. We showed that global surface area and intracranial volume, but not cortical thickness show substantial correlation of the overall intelligence (g-factor), thus affirming the association between cognitive abilities and brain organization [45, 46]. In line with previous observations, the strength of this association was moderate [47] and more notable at baseline. Schmitt et al. [48] have also reported that beyond the age of 10–11 years, the association between cortical thickness and intelligence is weak. As suggested by others, the relationship between brain structure and cognitive ability might be ever-changing and is likely to be influenced both by baseline brain structure and by its dynamic changes over time [49].

A key finding of the current study with important public health implications concerns the “lingering” influence of parental smoking and birth weight for brain structure in adolescence. Cigarette smoking in pregnant women has been associated with premature birth, low birth weight, stillbirth, asthma, learning and behavioural disability, and a predisposition to disease [50]. The mechanisms underlying the relation between perinatal exposure to smoking and brain structure are beyond the resolution of the available data in this study, but we note that maternal smoking has been associated with epigenetic modulation of birth weight [51]. There may be further mechanistic links as smoking has emerged as one of the most powerful epigenetic modulators amongst environmental exposures [52].

Alcohol and cannabis use were associated with accelerated cortical thinning and mild increase in cortical surface area and subcortical volumes. Our findings are generally in line with previous studies [27, 28] showing that even the mild substance use commonly encountered in general population is associated with measurable structural changes in the brain although the literature on the specific regions impacted is less consistent [53, 54]. Frontoparietal and cingulate cortices had the largest decrease in cortical thickness in relation to substance use and sensation seeking behaviour, possibly delineating the critical role of maturational changes in these regions in development of inhibitory control during adolescence [55].

Personality traits associated with anxiety and neuroticism were also associated with smaller surface area in adolescents. Similar results were obtained in young adults participating in the Human Connectome Project; in that study, neuroticism was negatively associated with cortical surface area in the left precentral, left superior parietal, left occipital and right superior temporal regions [25]. Some studies have suggested that the association between neuroticism and brain structure is sex-dependent [56]. Our results suggest that this may not be the case in this age-group when multiple other factors are simultaneously modelled. Intriguingly, conscientiousness had a negative cross-loading to the variate of subcortical volumes. Conscientiousness has shown positive associations with processing speed [57–60] but negative associations with fluid intelligence [61, 62], the latter being associated with larger subcortical volumes [63]. Although speculative, the negative cross-loading of conscientiousness with developmental change in subcortical volume may be aligned with proposal that high level of persistence and dutifulness may compensate for lower general abilities [61, 62].

The main strengths of this study include the large sample size, longitudinal design, and rich phenotyping of the IMAGEN cohort. We adopted a robust quality control procedure, where we used a longitudinal image analysis pipeline together with a two-level quality control process. Further, the analytic methods addressed several major issues in population neuroscience including analysis of high-dimensional data, stability, and reliability. Study limitations include the exclusive focus on atlas-based measures of brain structure, which provides a common framework for image analysis, but arguably limits the granularity of the data analysis. Structural measures are more reliable than other brain phenotypes but the lack of other brain phenotypes in the current study limits the generalizability of the findings to brain function or connectivity.

In summary, using multivariate statistical techniques, we found multiple reliable correlates of adolescent brain structure. Our study highlights the critical role for programmed biological processes such as indicated by sex, age, measures of physical growth, and intellectual functioning in brain development. Nevertheless, our findings also provide evidence for numerically smaller but statistically robust associations between brain structural phenotypes and modifiable social and environmental influences such as substance use, parental education, and life and perinatal events.

## Supplementary information

Supplemental Material

## Data Availability

Analysis code is available at https://github.com/AmirhosseinModabbernia/IMAGEN.
