# Supplementary material for: Linked patterns of biological and environmental covariation with brain structure in adolescence: a population-based longitudinal study
Source: Mol Psychiatry. 2020 May 22;26(9):4905–18. doi: 10.1038/s41380-020-0757-x (PMC7981783; doi:10.1038/s41380-020-0757-x)
Supplement: Supplementary file 1 — Supplemental Material [file 41380_2020_757_MOESM1_ESM.docx]

**Supplemental Material**

**Supplemental Methods**

1. **Participants**

Figure S1. Flowchart of participant selection for the current study

Table S1. Characteristics of the follow-up sample at the baseline and follow-up assessments

1. **IMAGEN Non-Imaging Variables**

Definition of variables and of the instruments used in the assessments

Table S2. Definition and assessment of non-imaging variables

Table S3. Percentage of missing data per non-imaging variable

1. **IMAGEN Neuroimaging Pipeline**

Table S4. Imaging measures derived from FreeSurfer

1. **Statistical Analyses: Sparse Canonical Correlation Analysis (sCCA)**

**4.1 General Principles**

**4.2 Definition of sCCA terms**

**4.3 Application of sCCA in the current study**

**4.3.1 Rationale**

**4.3.1 Choice of sparsity**

1. **Statistical Analyses: Code Availability**
2. **Statistical Analyses: Testing Reliability using the Redundancy-Reliability Score**

**Supplemental Results**

1. **Variable Distribution in the Baseline Sample and the Follow-up Sub-sample subsamples**

Table S5. Mean (standard deviation) of Neuroimaging Variables

1. **Sparse Canonical Correlation Analyses: Cortical Thickness**

Figure S2. Cortical Thickness at baseline: covariance explained by the canonical variates

Figure S3. Cortical Thickness developmental change: covariance explained by the canonical variates

Table S6. Cortical Thickness supplemental at baseline: Canonical weights (w) of the non-imaging variables

Table S7. Cortical Thickness supplemental at baseline: Canonical loadings (ρ) of the non-imaging variables

Table S8. Cortical Thickness supplemental at baseline: Canonical weights (w) of the imaging variables

Table S9. Cortical Thickness supplemental at baseline: Canonical loadings (ρ) of the imaging variables

Table S10. Cortical Thickness developmental change: Canonical weights (w) of the non-imaging variables

Table S11. Cortical Thickness developmental change: Canonical loadings (ρ) of the non-imaging variables

Table S12. Cortical Thickness developmental change: Canonical weights (w) of the imaging variables

Table S13. Cortical Thickness developmental change: Canonical loadings (ρ) of the imaging variables

1. **Sparse Canonical Correlation Analyses: Cortical Surface Area**

Figure S4. Cortical Surface Area at baseline: covariance explained by the canonical variates

Figure S5. Cortical Surface Ara developmental change: covariance explained by the canonical variates

Table S14. Cortical Surface Area supplemental at baseline: Canonical weights (w) of the non- imaging variables

Table S15. Cortical Surface Area supplemental at baseline: Canonical loadings (ρ) of the non-imaging variables

Table S16. Cortical Surface Area supplemental at baseline: Canonical weights (w) of the imaging variables

Table S17. Cortical Surface Area supplemental at baseline change: Canonical loadings (ρ) of the imaging variables

Table S18. Cortical Surface Area developmental change: Canonical weights (w) of the non- imaging variables

Table S19. Cortical Surface Area developmental change: Canonical loadings (ρ) of the non-imaging variables

Table S20. Cortical Surface Area developmental change: Canonical weights (w) of the imaging variables

Table S21. Cortical Surface Area developmental change: Canonical loadings (ρ) of the imaging variables

1. **Sparse Canonical Correlation Analyses: Subcortical Volumes**

Figure S6. Subcortical volume at baseline: covariance explained by the canonical variates

Figure S7. Subcortical volume developmental change: covariance explained by the canonical variates

Table S22. Subcortical Volumes supplemental at baseline: Canonical weights (w) of the non-imaging variables

Table S23. Subcortical Volumes supplemental at baseline: Canonical loadings (ρ) for the non-imaging variables

Table S24. Subcortical Volumes supplemental at baseline change: Canonical weights (w) of the imaging variables

Table S25. Subcortical Volumes supplemental at baseline: Canonical loadings (ρ) of the imaging variables

Table S26. Subcortical Volumes developmental change: Canonical weights (w) of the non-imaging variables

Table S27. Subcortical Volumes developmental change: Canonical loadings (ρ) of the non-imaging variables

Table S28. Subcortical Volumes developmental change: Canonical weights (w) of the imaging variables

Table S29. Subcortical Volumes developmental change: Canonical loadings (ρ) of the imaging variables

1. **Reliability and reproducibility**

Figure S8. Reliability of the results as a function of sample size and composition

Figure S9. Redundancy Reliability Scores

Table S30. Mean and standard deviations of supplemental Correlation coefficients for the first five modes in 500 train/test sets

Table S31. P-values for the first five SCCA modes after regressing out sex and age

Table S32. P-values of the first five SCCA modes of developmental change data after including variables only measured at baseline

1. **References**
2. **List of publications by the IMAGEN Consortium**

**Supplemental Methods**

1. **Participants**

The flowchart for participant selection from the IMAGEN cohort is shown in Figure S1. The non-imaging characteristics of the baseline and of the follow-up sample are shown in the Table 1 in the main text. The characteristics of the follow-up sample at their baseline and follow-up assessments are shown at Table S1.


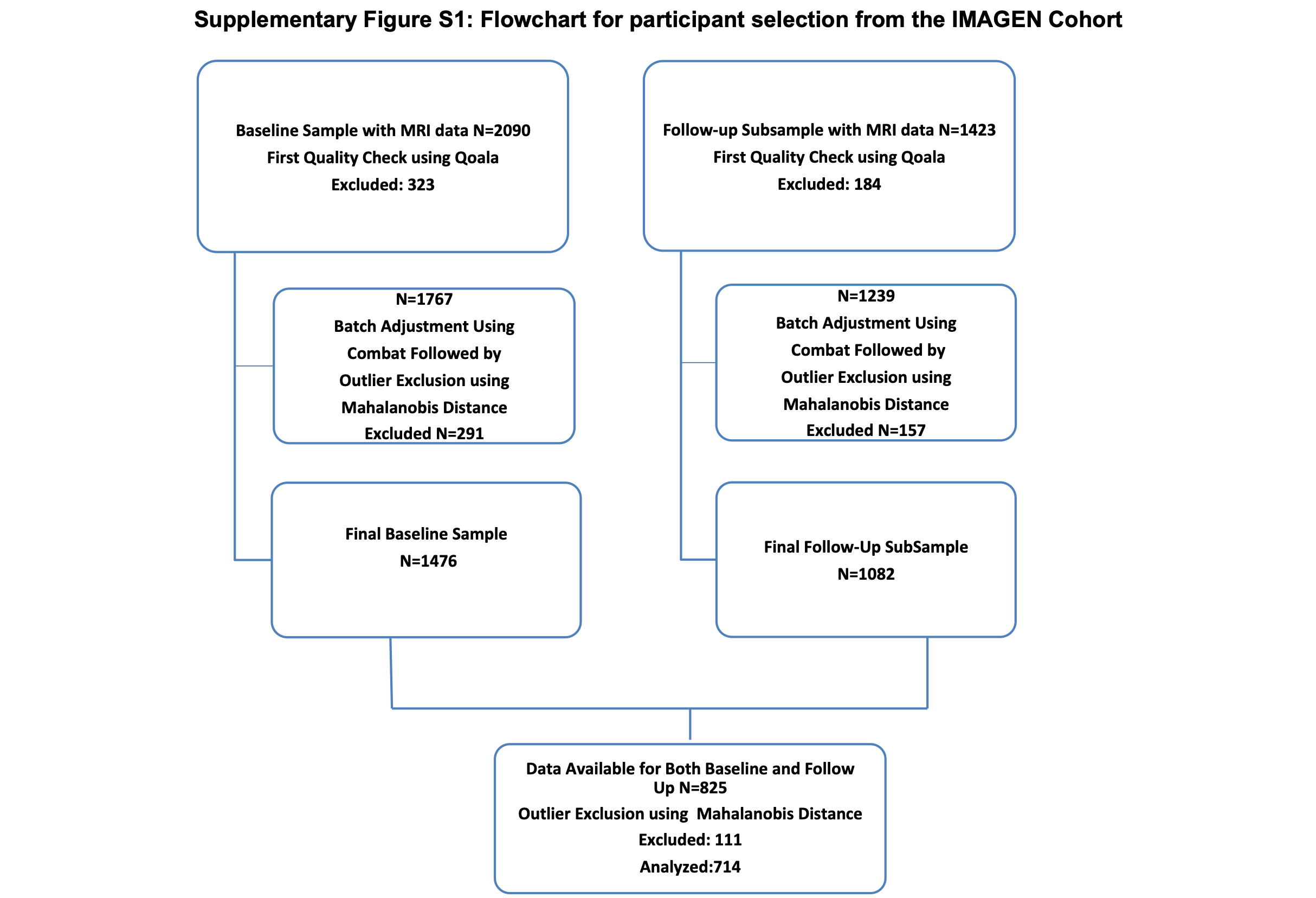


| **Table S1. Changes in the developmental change subsample (n=714) between their baseline and follow-up assessments** | | |
| --- | --- | --- |
| **Variable** | **Baseline Assessment** | **Follow-up Assessment** |
| **Youth Demographic and Anthropometric Features** | | |
| **Sex (female)** | 445 (62%) | - |
| **Age (years)*** | 14.45 (0.41) | 19.07 (0.75) |
| **Height (cm)*** | 167.32 (7.81) | 172.31 (9.14) |
| **Weight (kg)*** | 57.58 (10.29) | 66.61 (12.45) |
| **Body Mass Index*** | 20.5 (2.99) | 22.37 (3.47) |
| **Physical Development Scale** | 13.2 (2.2) | - |
| **Youth Perinatal Events** | | |
| **Birth Weight (grams)** | 3419 (553) | - |
| **Maternal Smoking During Pregnancy** | 76 (12%) | - |
| **Paternal Smoking During Pregnancy** | 108 (18%) | - |
| **Maternal Alcohol Use During Pregnancy** | 142 (23%) | - |
| **Maternal Medical Illness During Pregnancy** | 51 (8%) | - |
| **Pregnancy and/or Birth Complications** | 117 (19%) | - |
| **Breastfed** | 535 (86%) | - |
| **Youth Mental Health** | | |
| **Psychiatric Diagnosis*** | 79 (11%) | 99 (14%) |
| **Youth Cognitive Ability** | | |
| **General Intelligence (g-factor), Z-Score** | 0.17 (0.88) | - |
| **ESPAD: Average Grade**   - 1: C- - 2: C - 3: C+ - 4: B- - 5: B - 6: B+ - 7: A- - 8: A | 2 (0.3%)  10 (2%)  17 (3%)  18 (3%)  59 (9%)  178 (29%)  244 (39%)  92 (15%) | 9 (1%)  7 (1%)  21 (3%)  28 (4%)  53 (9%)  192 (31%)  210 (34%)  99 (16%) |
| **ESPAD: Truancy*** | 3.99 (1.69) | 4.19 (1.86) |
| **Youth Personality and Temperament** | | |
| **NEO: Neuroticism*^1^** | 1.91 (0.57) | 1.71 (0.66) |
| **NEO: Extroversion** | 2.43 (0.44) | 2.41 (0.45) |
| **NEO: Openness*** | 2.24 (0.49) | 2.36 (0.51) |
| **NEO: Agreeableness*** | 2.38 (0.4) | 2.56 (0.42) |
| **NEO: Conscientiousness*** | 2.36 (0.56) | 2.51 (0.61) |
| **DAWBA Social Aptitude Scale*** | 24.61 (5.6) | 25.61 (6.25) |
| **TCI: Novelty Seeking*** | 110.63 (10.47) | 107.12 (10.76) |
| **Youth Substance Risk and Use** | | |
| **SURPS: Anxiety Sensitivity*** | 2.26 (0.46) | 2.35 (0.46) |
| **SURPS: Hopelessness** | 1.87 (0.42) | 1.86 (0.47) |
| **SURPS: Impulsivity*** | 2.39 (0.44) | 2.19 (0.41) |
| **SURPS: Sensation Seeking** | 2.74 (0.54) | 2.78 (0.54) |
| **ESPAD Frequency of ESPAD Frequency of Lifetime Smoking***   - 0: 0 - 1: 1-2 times - 2: 3-5 times - 3: 6-9 times - 4: 10-19 times - 5: 20-39 times - 6: 40 or more times | 459 (74%)  86 (14%)  19 (3%)  15 (2%)  16 (3%)  10 (2%)  15 (2%) | 196 (32%)  77 (12%)  61 (10%)  30 (5%)  45 (8%)  28 (4%)  182 (29%) |
| **ESPAD: Smoking the preceding 30 days***   - 0: Not at all - 1: less than 1 cigarette per week - 2: less than 1 cigarette per day - 3: 1-5 cigarettes per day - 4: 6-10 cigarettes per day - 5: 11-20 cigarettes per day - 6: more than 20 cigarettes per day | 571 (92%)  26 (4%)  9 (1%)  8 (1%)  3 (0.5%)  3 (0.5%)  0 (0%) | 384 (62%)  65 (10%)  42 (7%)  49 (8%)  45 (7%)  30 (5%)  4 (1%) |
| **ESPAD: Lifetime Alcohol Use***   - 0: 0 - 1: 1-2 times - 2: 3-5 times - 3: 6-9 times - 4: 10-19 times - 5: 20-39 times - 6: 40 or more times | 135 (22%)  161 (26%)  127 (21%)  86 (14%)  68 (11%)  24 (4%)  17 (3%) | 15 (2%)  15 (2%)  24 (4%)  37 (6%)  67 (11%)  106 (17%)  355 (57%) |
| **ESPAD:** **Alcohol Use in the preceding 30 days***   - 0: 0 - 1-2 times - 3-5 times - 6-9 times - 10-19 times - 20-39 times - 40 or more times | 325 (53%)  218 (35%)  46 (7%)  16 (3%)  10 (2%)  2 (0.3%)  1 (0.2%) | 54 (9%)  194 (31%)  153 (25%)  113 (18%)  72 (12%)  20 (3%)  13 (2%) |
| **ESPAD Frequency of Lifetime Cannabis use ***   - 0: 0 - 1: 1-2 times - 2: 3-5 times - 3: 6-9 times - 4: 10-19 times - 5: 20-39 times - 6: 40 or more times | 592 (96%)  16 (3%)  3 (0.5%)  2 (0.3%)  0 (0%)  0 (0%)  3 (0.5%) | 320 (52%)  65 (11%)  48 (8%)  40 (6%)  35 (6%)  32 (5%)  78 (13%) |
| **ESPAD: Cannabis Use in the preceding 30 days***   - 0: 0 - 1: 1-2 times - 2: 3-5 times - 3: 6-9 times - 4: 10-19 times - 5: 20-39 times - 6: 40 or more times | 603 (98%)  11 (2%)  0 (0%)  0 (0%)  0 (0%)  0 (0%)  2 (0.3%) | 347 (56%)  116 (19%)  51 (8%)  33 (5%)  33 (5%)  22 (4%)  16 (3%) |
| **Youth Social and Family Circumstances** | | |
| **LEQ: Number of Negative Life Events*** | 5.66 (3.03) | 3.19 (2.29) |
| **LEQ: Family*** | 0.24 (0.22) | 0.13 (0.17) |
| **LEQ: Accident*** | 0.51 (0.27) | 0.35 (0.26) |
| **LEQ: Sexuality*** | 0.28 (0.18) | 0.35 (0.21) |
| **LEQ: Autonomy*** | 0.53 (0.17) | 0.6 (0.19) |
| **LEQ: Deviance*** | 0.24 (0.23) | 0.09 (0.19) |
| **LEQ: Relocation*** | 0.45 (0.33) | 0.11 (0.19) |
| **LEQ: Distress*** | 0.28 (0.19) | 0.21 (0.18) |
| **LEQ: Other*** | 0.34 (0.28) | 0.29 (0.24) |
| **ESPAD: Victim of Bullying*** | 0.2 (0.4) | 0.04 (0.21) |
| **ESPAD: Perpetrator of Bullying*** | 0.07 (0.26) | 0.01 (0.13) |
| **DAWBA: Family Stressors: Financial/Housing** | 0.65 (1.03) | 0.59 (1.02) |
| **DAWBA: Family Stressors: Work/Pressure*** | 1.11 (1.09) | 1.01 (1.08) |
| **DAWBA: Family Stressors: Illness** | 0.51 (0.9) | 0.55 (0.94) |
| **DAWBA: Family Stressors Relationships/Addiction** | 0.39 (0.73) | 0.39 (0.7) |
| **DAWBA: Child Experience: Affirmation** | 10.9 (1.35) | 10.93 (1.36) |
| **DAWBA: Child Experience: Discipline*** | 3.35 (1.58) | 2.96 (1.5) |
| **DAWBA: Child Experience: Rules** | 4.64 (1.27) | 4.67 (1.26) |
| **DAWBA: Living With Both Parents*** | 620 (87%) | 554 (88%) |
| **FIGS: Positive Family History of Psychiatric Disorders** | 129 (18%) | - |
| **Parental Characteristics** | | |
| **ESPAD: Maternal Education Level**   - GCSEs or CSEs or below - NVQ or GNVQ - A levels or a BTEC national diploma - Advanced diploma - Bachelor degree - Professional Qualifications (Master’s degree and above) | 90 (13%)  112 (16%)  111 (16%)  100 (15%)  175 (26%)  91 (13%) | -  -  -  -  -  - |
| **ESPAD: Paternal Education Level**   - GCSEs or CSEs or below - NVQ or GNVQ - A levels or a BTEC national diploma - Advanced diploma - Bachelor degree - Professional Qualifications (Master’s degree and above) | 113 (17%)  109 (16%)  86 (13%)  78 (12%)  168 (25%)  125 (18%) | -  -  -  -  -  - |
| * Denotes differences between baseline and follow-up at P<0.05 uncorrected; Continuous variables are shown as mean (standard deviation); categorical variables are shown as number (percentage;%); follow-up LEQ values are mean number of life events happening after the last visit. ESPAD= European School Survey Project on Alcohol and Other Drugs; DAWBA=Development and Well-being Assessment; FIGS: Family Interview for Genetic Studies; LEQ=Life Events Questionnaire; NEO= NEO-Five Factor Personality Inventory; SURPS=Substance Use Risk Profile Scale; TCI=Temperament and Character Inventory; WISC-IV=Wechsler Intelligence Scale for Children-IV; GCSE=General Certificate of Secondary Education; CSE= Certificate of Secondary Education; GNVC= General National Vocational Qualification; NVQ= National Vocational Qualification; BTEC= Business and Technology Education Council; A levels=Advance Level Qualification. Details on each variable in Supplemental Table S2 and S3. | | |

1. **IMAGEN Non-Imaging Variables**

Details of the assessment instruments used in IMAGEN are provided in Table S2. These assessments and the quality control procedures have been described in previous publications from the Consortium (Schumann et al., 2010). Youth responses were coded with regards to their validity for quality control purposes. Responses that were recorded when a youth was distracted or watched by another person were excluded as invalid. The percentage of missing values for each variable in each sample is shown in Table S3.

With the exception of perinatal events, all assessments were conducted at baseline and at the follow-up assessment at age 19 years. Participants had an interim assessment at age 16 years which did not include imaging.

| **Table S2. Definition and Assessment of non-imaging Variables** | | | |
| --- | --- | --- | --- |
| **Variable Class** | **Measures** | **Instrument** | **Description of the Variables Used** |
| **Demographic and Anthropometric Measures** | Sex, Age, Weight, Height, Body Mass Index, pubertal developmental stage | The Pubertal Development Scale (PDS) includes five puberty-related questions for each sex | Sex (Male, Female), Age in years, Weight in kg and Height in cm.  Body Mass Index was calculated as weight in kg divided by the squared height in cm.  For the PDS, we calculated Z-scores for sum of five items for each sex |
| **Perinatal Events** | Pregnancy and birth risks and complications | The Pregnancy and Birth Questionnaire used to assess the presence or absence of 7 conditions: maternal prenatal smoking, paternal prenatal smoking, maternal prenatal alcohol use, pregnancy complications, maternal medical conditions during pregnancy, birth weight, and breastfeeding | Seven binarised scores for each condition |
| **Mental Health** | Psychiatric Diagnosis | Either an ICD-10 or DSM-IV-TR Psychiatric Diagnosis which instrument | Binarised to denote the presence or absence of a psychiatric disorder |
| **Cognitive Ability** | Intelligence g-Factor | Wechsler Intelligence Scale for Children-IV (WISC-IV):  Six subscales (block design, vocabulary, similarities, matrix reasoning, and digit span forward and backward) | We applied principal component analysis on the six subscales; the score on first principal component as a measure of the g-Factor. |
|  | Academic performance | European School Survey Project on Alcohol and Other Drugs (ESPAD) includes one item on academic performance and 3 items on truancy | Academic Performance: Level of overall school performance in the preceding academic term  Truancy: Days school missed in the preceding month because of illness, skipping off or any other reason |
| **Personality** | Personality Dimensions | The NEO-Five Factor Personality Inventory^1^ has 60 items, twelve questions for each of neuroticism, extroversion, openness to experience, conscientiousness, agreeableness | Individual subscale scores for neuroticism, extroversion, openness to experience, conscientiousness, agreeableness |
|  | Temperament | The Temperament and Character Inventory (TCI) | Only the score for Novelty Seeking was used |
|  | Social Aptitude | The Development and Well-being Assessment (DAWBA) includes 10 questions on socially appropriate behavior which constitute the Social Aptitude Scale (SAS) | Total SAS score |
| **Substance Risk and Use** | Substance Use | European School Survey Project on Alcohol and Other Drugs (ESPAD) has multiple questions to assess cannabis, alcohol and tobacco use | Smoking was assessed in terms of lifetime number of occasions of use and number of cigarettes in the preceding 30 days; Alcohol was assessed in terms of number of occasions of use over the lifetime and in the preceding 30 days; Cannabis use was assessed in terms of number of occasions of use over the lifetime and in the preceding 30 days |
|  | Substance Use Risk | Substance Use Risk Profile Scale (SURPS) has 23 items with questions with 7 questions for hopelessness (negative thinking), 6 questions for sensation seeking, and 5 questions each for impulsivity and anxiety sensitivity | Individual subscale scores for Anxiety sensitivity, hopelessness, impulsivity, sensation seeking |
| **Family and Social Circumstances** | Life events | The Life Events Questionnaire (LEQ) includes 39 items about frequency and type of life events in 8 domains:  --Family/Parents (comprising parental divorce, parental discord, parental remarriage, parental alcohol abuse, family financial difficulties)  - Accident/ Illness (comprising accident/illness, given medication by a physician, death in family, serious accident or illness)  - Sexuality (comprising falling in love, starting or ending a relationship, having first sexual experience, having a gay experience, pregnancy)  - Autonomy (events relating to independence including starting college, a hobby or new friends)  - Deviance (getting in trouble, at school or the law, stealing)  - Relocation (change of school or residence)  - Distress (comprising Face breaking out with pimples, starting to see a therapist, thinking about suicide, running away from home, getting poor grades at school, and gaining a lot of weight)  - Events not otherwise covered (sibling moving out, meeting a teacher who he/she likes a lot, finding a religion) | Mean frequency of life events in 8 domains and total number of negative life events in the preceding year? |
|  | Bullying | The ESPAD contains 6 items on being a bullying perpetrator and 6 items on being a victim of bullying | Sum of the 6 items on being a perpetrator and sum of the 6 items on being a victim |
|  | Family stressors | The DAWBA includes 16 items about family stressors in 4 domains  -Socioeconomic/housing  -Work/pressure  -Illness  -Relationships/addiction | Individual score from each of the 4 domains |
|  | Childhood experience of family life | The DAWBA includes 21 questions about child experience of family life | Individual domain scores for Affirmation, Rules, and Discipline |
|  | Living with parents | The DAWBA includes a single question as to whether the child lives with one family or has any other living arrangement | Binarised item to denote living with one family vs any other living arrangement |
|  | Family history of psychiatric illness | Psychiatric illness in the first degree relatives assessed with the Family Interview for Genetic Studies (FIGS) | Binarised to denote the absence or presence of any psychiatric diagnosis in first-degree relatives |
| **Parental Characteristics** | Parental Education | The ESPAD provides a single score for paternal and another for maternal education on six levels:  1 General Certificate of Secondary Education (GCSE) or Certificate of Secondary Education (CSE) or below  2 General National Vocational Qualification (GNVQ) or National Vocational Qualification (NVQ)  3 Advanced Level Qualification (A levels) or a Business and Technology Education Council (BTEC) national diploma  4 Advanced diploma  5 Bachelor degree e.g. BA, BSc  6 Professional qualification e.g. MSc, PhD, MD | A single score for each parent |

| **Table S3. Percentage of missing data per non-imaging variable** | | |
| --- | --- | --- |
| **Variable** | **Baseline**  **Sample** | **Developmental**  **Change Sub-sample** |
| **Sex** | 0 | 0 |
| **Age** | 0 | 0 |
| **Height** | 0 | 0 |
| **Weight** | 0 | 0 |
| **Body Mass Index** | 0 | 0 |
| **Pubertal Development Scale** | 10.5 | 4 |
| **Birth Weight** | 16.9 | 6.4 |
| **Maternal Smoking During Pregnancy** | 17.4 | 6.8 |
| **Paternal Smoking During Pregnancy** | 18 | 7.0 |
| **Maternal Alcohol Use During Pregnancy** | 17.8 | 7.1 |
| **Maternal Medical Illness During Pregnancy** | 17.2 | 6.7 |
| **Pregnancy and/or Birth Complications** | 16.9 | 6.4 |
| **Breastfed** | 16.9 | 6.4 |
| **Youth Psychiatric Diagnosis** | 0.5 | 0.14 |
| **WISC-IV: Intelligence g-Factor** | 0 | 0 |
| **ESPAD: Average Grade** | 14.6 | 6.5 |
| **ESPAD: Truancy** | 14.5 | 10 |
| **NEO: Neuroticism** | 10.2 | 0.8 |
| **NEO: Extroversion** | 10.2 | 0.8 |
| **NEO: Openness** | 10.2 | 0.8 |
| **NEO: Agreeableness** | 10.2 | 0.8 |
| **NEO: Conscientiousness** | 10.2 | 0.8 |
| **DAWBA: Social Aptitude Scale** | 0.9 | 5.8 |
| **TCI: Novelty Seeking** | 10.1 | 3.7 |
| **SURPS: Anxiety Sensitivity** | 10.1 | 3.4 |
| **SURPS: Hopelessness** | 10.1 | 3.4 |
| **SURPS: Impulsivity** | 10.1 | 3.4 |
| **SURPS: Sensation Seeking** | 10.1 | 3.4 |
| **ESPAD: ESPAD Frequency of ESPAD Frequency of Lifetime Smoking** | 14.6 | 6.5 |
| **ESPAD: Smoking in the preceding 30 days** | 14.6 | 6.5 |
| **ESAPD: Lifetime Alcohol Use** | 14.8 | 6.6 |
| **ESPAD: Alcohol use in the preceding 30 days** | 14.8 | 6.6 |
| **ESPAD Frequency of Lifetime Cannabis use** | 15 | 6.6 |
| **ESPAD: Cannabis Use in the preceding 30 days** | 15 | 6.6 |
| **LEQ: Total Number of Negative Life Events** | 1.6 | 0.03 |
| **LEQ: Family** | 1.6 | 0.03 |
| **LEQ: Accident** | 1.6 | 0.03 |
| **LEQ: Sexuality** | 1.6 | 0.03 |
| **LEQ: Autonomy** | 1.6 | 0.03 |
| **LEQ: Deviance** | 1.6 | 0.03 |
| **LEQ: Relocation** | 1.6 | 0.03 |
| **LEQ: Distress** | 1.6 | 0.03 |
| **LEQ: Other** | 1.6 | 0.03 |
| **ESPAD: Victim of Bullying** | 15.1 | 0.14 |
| **ESPAD: Perpetrator of Bullying** | 15.1 | 0.14 |
| **DAWBA: Family Stressors Socioeconomic/Housing** | 0.6 | 1.5 |
| **DAWBA: Family Stressors Work/Pressure** | 0.6 | 1.5 |
| **DAWBA: Family Stressors Illness** | 0.6 | 1.5 |
| **DAWBA: Family Stressors Relationship/Addiction** | 0.6 | 1.5 |
| **DAWBA: Child Experience Affirmation** | 0.6 | 1.5 |
| **DAWBA: Child Experience Discipline** | 0.6 | 1.5 |
| **DAWBA: Child Experience Rules** | 0.6 | 1.5 |
| **DAWBA: Living with both Parents** | 0.6 | 1.5 |
| **FIGS: Family History of Psychiatric Disorders** | 0.4 | 0.14 |
| **ESPAD: Maternal Education Level** | 5.3 | 2.4 |
| **ESPAD: Paternal Education Level** | 5.3 | 2.4 |
| ESPAD= European School Survey Project on Alcohol and Other Drugs; DAWBA=Development and Well-being Assessment; FIGS: Family Interview for Genetic Studies; LEQ=Life Events Questionnaire; NEO=NEO-Five Factor Personality Inventory; SURPS=Substance Use Risk Profile Scale; TCI=Temperament and Character Inventory; WISC-IV=Wechsler Intelligence Scale for Children-IV | | |

**3. IMAGEN Neuroimaging Pipeline**

High-resolution T_1_-weighted images were obtained using a 3D T1-weighted sequence with a1.1 mm isotropic voxel size, based on the ADNI protocol (<http://www.loni.ucla.edu/ADNI/Cores/index.shtml>). There were eight sites (London, Nottingham, Dublin, Mannheim, Dresden, Berlin, Hamburg, and Paris) using 3T MRI systems from 4 different manufacturers (Siemens: 4 sites, Philips: 2 sites, General Electric: 1 site, and Bruker: 1 site). In addition, to the standard IMAGEN procedures^2^ we applied a validated automatic quality control algorithm (Qoala-T; (https://github.com/Qoala-T/QC)^3^ to cross-sectionally preprocessed MRI scans to exclude low quality images (Figure S1). Subsequently, we used an automatic robust longitudinal processing pipeline^4^ to extract reliable estimates of cortical thickness, surface area, and subcortical volumes using Freesurfer version 6.0 (<https://surfer.nmr.mgh.harvard.edu/>). Table S4 lists the imaging derived imaging variables.

| **Table S4. Imaging measures derived from FreeSurfer** | |
| --- | --- |
| **Measure** | **Regions** |
| **Cortical Thickness** | left_bankssts, left_caudalanteriorcingulate, left_caudalmiddlefrontal, left_cuneus, left_entorhinal, left_fusiform, left_inferiorparietal, left_inferiortemporal, left_isthmuscingulate, left_lateraloccipital, left_lateralorbitofrontal, left_lingual, left_medialorbitofrontal, left_middletemporal, left_parahippocampal, left_paracentral, left_parsopercularis, left_parsorbitalis, left_parstriangularis, left_pericalcarine, left_postcentral, left_posteriorcingulate, left_precentral, left_precuneus, left_rostralanteriorcingulate, left_rostralmiddlefrontal, left_superiorfrontal, left_superiorparietal, left_superiortemporal, left_supramarginal, left_frontalpole, left_temporalpole, left_transversetemporal, left_insula, right_bankssts, right_caudalanteriorcingulate, right_caudalmiddlefrontal, right_cuneus, right_entorhinal, right_fusiform, right_inferiorparietal, right_inferiortemporal, right_isthmuscingulate, right_lateraloccipital, right_lateralorbitofrontal, right_lingual, right_medialorbitofrontal, right_middletemporal, right_parahippocampal, right_paracentral, right_parsopercularis, right_parsorbitalis, right_parstriangularis, right_pericalcarine, right_postcentral, right_posteriorcingulate, right_precentral, right_precuneus, right_rostralanteriorcingulate, right_rostralmiddlefrontal, right_superiorfrontal, right_superiorparietal, right_superiortemporal, right_supramarginal, right_frontalpole, right_temporalpole, right_transversetemporal, right_insula |
| **Cortical Surface Area** | left_bankssts, left_caudalanteriorcingulate, left_caudalmiddlefrontal, left_cuneus, left_entorhinal, left_fusiform, left_inferiorparietal, left_inferiortemporal, left_isthmuscingulate, left_lateraloccipital, left_lateralorbitofrontal, left_lingual, left_medialorbitofrontal, left_middletemporal, left_parahippocampal, left_paracentral, left_parsopercularis, left_parsorbitalis, left_parstriangularis, left_pericalcarine, left_postcentral, left_posteriorcingulate, left_precentral, left_precuneus, left_rostralanteriorcingulate, left_rostralmiddlefrontal, left_superiorfrontal, left_superiorparietal, left_superiortemporal, left_supramarginal, left_frontalpole, left_temporalpole, left_transversetemporal, left_insula, right_bankssts, right_caudalanteriorcingulate, right_caudalmiddlefrontal, right_cuneus, right_entorhinal, right_fusiform, right_inferiorparietal, right_inferiortemporal, right_isthmuscingulate, right_lateraloccipital, right_lateralorbitofrontal, right_lingual, right_medialorbitofrontal, right_middletemporal, right_parahippocampal, right_paracentral, right_parsopercularis, right_parsorbitalis, right_parstriangularis, right_pericalcarine, right_postcentral, right_posteriorcingulate, right_precentral, right_precuneus, right_rostralanteriorcingulate, right_rostralmiddlefrontal, right_superiorfrontal, right_superiorparietal, right_superiortemporal, right_supramarginal, right_frontalpole, right_temporalpole, right_transversetemporal, right_insula |
| **Subcortical Volumes** | Left.Lateral.Ventricle, Left.Cerebellum.Cortex, Left.Thalamus.Proper, Left.Caudate, Left.Putamen, Left.Pallidum, Left.Hippocampus, Left.Amygdala, Left.Accumbens.area, Left.VentralDC , right.Lateral.Ventricle, Right.Cerebellum.Cortex, Right.Thalamus.Proper, Right.Caudate, Right.Putamen, Right.Pallidum, Right.Hippocampus, Right.Amygdala, Right.Accumbens.area, Right.VentralDC |
| **Global Measures** | Mean Thickness (left and right), Total Intracranial Volume (TIV), Total Surface Area |

1. **Statistical Analyses: Sparse Canonical correlation analysis**
   1. **Sparce Canonical correlation analysis: General Principles**

Canonical correlation analysis (CCA) is a general method for finding relationships between two multivariate sets of variables (X and Y datasets), all measured in the same individuals. It can be considered as a generalization of multiple linear regression. CCA aims to find those linear combinations (pairs of canonical variates or otherwise modes) of variables in the X dataset and variables in the Y dataset that best express the maximal correlation (i.e., canonical correlation) between the two datasets. Suppose we wished to examine the relationship between the variables in the X dataset (variables x_1_, x_2,_…., x_p_) and the Y dataset (variables y_1_, y_2_,...., y_q_). The correlations between the canonical variates are the canonical correlations. A typical CCA would progress in *n* iterative steps each forming two linear combinations following the generic formula: W_n_ = a_1n_x_1_ + a_2n_x_2_ +…. + a_np_x_p_ and V_n_ = b_1n_y_1_ + b_1n_y_2_ +….+ b_1nq_y_q_ , such that the correlation C_n_ of W_n_ and V_n_ is maximum and the correlations between (W_n_, W _n+1_) and (V_n_, V_n+1_) are zero.

In recent years, sparse CCA (sCCA) has been developed to address the drawbacks of the conventional approach. First, sCCA does not assume that the variables in the datasets are uncorrelated. Second, in conventional CCA, all weights contributing to any significant relation between datasets are non-zero and is therefore not possible to make inferences about individual variables. By contrast, in sCCA some of the elements of the canonical vectors are estimated as exactly zero using penalty functions. The canonical variates then only depend on the subset of the variables with non-zero elements of the estimated canonical vectors. This renders sCCA results easier to interpret when dealing with large datasets. Finally, sCCA allows the investigation of datasets possessed of more features than samples, which is often the case in neuroimaging.

**4.2 Terms used in sCCA**

*Canonical weights*: Canonical weights are equivalent of regression beta coefficients that are used to make a linear combination of variables in each dataset

*Canonical variates*: Canonical variates are the latent variables created by the linear combination of variables in each canonical mode

*Canonical modes*: Canonical modes refers to each pair of canonical variates

Canonical loading: Canonical loadings are the correlation between each variable and its corresponding canonical variate

*Canonical cross-loading*: Canonical cross-loadings are the correlation between each variable and the opposite canonical variate

- 1. **Application of sCCA in the current study**

**4.3.1 Rationale**:

Partial least squares (PLS) and canonical correlation analysis (CCA) are two of the most commonly used models for multivariate analyses. PLS seeks to maximize the covariance of the projected dimensions with the original variables of the “dependent variables” dataset, whereas CCA maximizes the correlation between the latent variables identified on each dataset and is thus more suitable for the purpose of our study.

We consider the use of CCA in the current study advantageous because it provides information about the order, direction and strength of association between variables.^5, 6^

**4.3.2 sCCA implementation**

Non-imaging and neuroimaging variables were standardized to a mean of 0 and a standard deviation of 1 before being entered into the sCCA models^7, 8^. We then followed standard procedures to identify the optimal sparsity parameters for each sCCA model. For each analysis, we computed the sparse parameters by running the sCCA with a range of candidate values (from 1/√p to 1, at 10 increments, where p is the number of features in that view of the data) for each imaging and non-imaging dataset, and then fitted the resulting models. We selected the optimal sparse criteria combination based on the parameters that corresponded to the values of the model that maximized the sCCA correlation value. We then computed the optimal sCCA model and determined its significance based on exact P-values calculated from 1000 random permutations. We permuted the imaging data 1000 times and recalculated the canonical correlation coefficients between imaging variables and non-imaging variables in the permuted dataset after rerunning the sCCA algorithm for each permutation. The exact p-value was defined as the number of permutations that resulted in an equal or higher correlation than the original data divided by the total number of permutations. Because we implemented multiple sCCA models throughout the manuscript, significance of each mode was further adjusted using false discovery correction (FDR). In addition, statistically significant modes were tested for reliability and reproducibility (described below) and only models that survived these analyses are reported. For significant sCCA mode, we report weights and loadings of the contributing variables if these are at least of small effect (>|0.1|) according to current standards^9^.

1. **Code Availability**

[**https://github.com/AmirhosseinModabbernia/IMAGEN**](https://github.com/AmirhosseinModabbernia/IMAGEN)

1. **Reliability analyses: Redundancy-Reliability Score**

For each Sparse Canonical Correlation Analysis (supplemental), we calculated the redundancy reliability (RR) scores following our prior work^10^ to quantify the reliability of the variable-to-variate correlation across 500 random subsets of the original sample. The RR score was calculated as:

$$\mathrm{RR}^{j}=\frac{\left| \mathrm{corr}\left( \rho_{W}^{\mathrm{TS}}, \rho_{W}^{j} \right) \right|+\left| \mathrm{corr}\left( \rho_{V}^{\mathrm{TS}}, \rho_{V}^{j} \right) \right|}{2}$$

where W denotes imaging-variable to non-imaging variate. V denotes non-imaging-variable to imaging variate.

$\rho_{W}^{j}$ are the variable-to-variate Pearson’s correlation between imaging variables and the non-imaging variate of the subset j

$\rho_{V}^{j}$ are the variable-to-variate Pearson’s correlation between non-imaging variables and the imaging variate of the subset j

$\rho_{W}^{\mathrm{TS}}$ are the mean variable-to-variate Pearson’s correlation coefficient between imaging variables and the non-imaging variate of the 500 subsets.

**Supplemental Results**

1. **Variable Distribution in the Baseline Sample and the Follow-up Sub-sample subsamples**

The distribution of the non-imaging variables per sample is presented in the main text. Supplemental Table S5 shows the mean and standard deviation of each imaging variable in the baseline sample and the follow-up and change subsamples.

| **Table S5. Mean and (standard deviation) of the imaging variables** | | | | |
| --- | --- | --- | --- | --- |
| **Imaging Variable** | | **Total**  **Sample**  **N=1476** | **Developmental Change**  **Subsample**  **N=714** | |
|  |  |  | **At baseline Assessment** | **At follow-up Assessment** |
| **Global Measures** | | | | |
| Mean cortical thickness-Right, mm | | 2.75(0.08) | 2.77(0.08) | 2.64(0.07) |
| Mean cortical thickness-Left, mm | | 2.7 (0.08) | 2.76(0.08) | 2.64(0.08) |
| Total Surface area, mm^2^ | | 181074.1  (15905.45) | 179217.1  (14811.77) | 175326.2  (14599.9) |
| Total intracranial volume, mm^3^ | | 1530240  (137074.6) | 1519667.88  (132068.71) | 1519667.88  (132068.71) |
| **Cortical Thickness** | | | | |
| lh_bankssts | | 2.77(0.16) | 2.8(0.16) | 2.63(0.14) |
| lh_caudalanteriorcingulate | | 2.95(0.23) | 2.98(0.23) | 2.83(0.22) |
| lh_caudalmiddlefrontal | | 2.83(0.12) | 2.85(0.12) | 2.67(0.12) |
| lh_cuneus | | 2.13(0.14) | 2.14(0.14) | 2.07(0.13) |
| lh_entorhinal | | 3.5(0.3) | 3.54(0.27) | 3.59(0.28) |
| lh_fusiform | | 2.94(0.11) | 2.95(0.11) | 2.88(0.11) |
| lh_inferiorparietal | | 2.72(0.11) | 2.74(0.1) | 2.56(0.1) |
| lh_inferiortemporal | | 2.93(0.15) | 2.95(0.14) | 2.89(0.13) |
| lh_isthmuscingulate | | 2.62(0.21) | 2.64(0.2) | 2.52(0.19) |
| lh_lateraloccipital | | 2.39(0.11) | 2.41(0.11) | 2.3(0.11) |
| lh_lateralorbitofrontal | | 2.91(0.13) | 2.92(0.12) | 2.81(0.11) |
| lh_lingual | | 2.29(0.12) | 2.3(0.12) | 2.26(0.11) |
| lh_medialorbitofrontal | | 2.73(0.15) | 2.73(0.15) | 2.6(0.14) |
| lh_middletemporal | | 3.06(0.14) | 3.09(0.13) | 2.98(0.13) |
| lh_parahippocampal | | 3.04(0.26) | 3.07(0.26) | 3.02(0.26) |
| lh_paracentral | | 2.7(0.14) | 2.72(0.14) | 2.59(0.13) |
| lh_parsopercularis | | 2.89(0.12) | 2.91(0.12) | 2.77(0.12) |
| lh_parsorbitalis | | 2.99(0.18) | 3.02(0.18) | 2.89(0.17) |
| lh_parstriangularis | | 2.77(0.13) | 2.79(0.13) | 2.65(0.13) |
| lh_pericalcarine | | 1.84(0.14) | 1.84(0.14) | 1.82(0.13) |
| lh_postcentral | | 2.34(0.12) | 2.35(0.12) | 2.25(0.11) |
| lh_posteriorcingulate | | 2.74(0.15) | 2.76(0.16) | 2.62(0.15) |
| lh_precentral | | 2.81(0.11) | 2.84(0.1) | 2.74(0.1) |
| lh_precuneus | | 2.68(0.11) | 2.69(0.11) | 2.55(0.1) |
| lh_rostralanteriorcingulate | | 3.14(0.21) | 3.16(0.21) | 3.04(0.21) |
| lh_rostralmiddlefrontal | | 2.63(0.11) | 2.65(0.11) | 2.49(0.1) |
| lh_superiorfrontal | | 3.03(0.12) | 3.05(0.12) | 2.87(0.11) |
| lh_superiorparietal | | 2.46(0.12) | 2.47(0.11) | 2.32(0.11) |
| lh_superiortemporal | | 3.06(0.14) | 3.08(0.14) | 2.97(0.13) |
| lh_supramarginal | | 2.84(0.12) | 2.87(0.11) | 2.69(0.11) |
| lh_frontalpole | | 3.04(0.25) | 3.06(0.25) | 2.9(0.23) |
| lh_temporalpole | | 3.66(0.31) | 3.73(0.28) | 3.72(0.27) |
| lh_transversetemporal | | 2.76(0.2) | 2.77(0.19) | 2.71(0.17) |
| lh_insula | | 3.29(0.14) | 3.3(0.13) | 3.2(0.12) |
| rh_bankssts | | 2.88(0.16) | 2.9(0.16) | 2.73(0.15) |
| rh_caudalanteriorcingulate | | 2.76(0.22) | 2.78(0.22) | 2.65(0.21) |
| rh_caudalmiddlefrontal | | 2.8(0.12) | 2.82(0.12) | 2.63(0.11) |
| rh_cuneus | | 2.16(0.13) | 2.17(0.13) | 2.1(0.12) |
| rh_entorhinal | | 3.61(0.33) | 3.66(0.31) | 3.7(0.31) |
| rh_fusiform | | 2.97(0.11) | 2.99(0.11) | 2.93(0.11) |
| rh_inferiorparietal | | 2.74(0.11) | 2.76(0.1) | 2.59(0.1) |
| rh_inferiortemporal | | 3.01(0.13) | 3.03(0.13) | 2.93(0.12) |
| rh_isthmuscingulate | | 2.67(0.2) | 2.68(0.21) | 2.57(0.19) |
| rh_lateraloccipital | | 2.46(0.11) | 2.47(0.11) | 2.37(0.11) |
| rh_lateralorbitofrontal | | 2.89(0.13) | 2.91(0.12) | 2.78(0.12) |
| rh_lingual | | 2.33(0.12) | 2.33(0.12) | 2.29(0.12) |
| rh_medialorbitofrontal | | 2.76(0.15) | 2.77(0.14) | 2.64(0.14) |
| rh_middletemporal | | 3.13(0.13) | 3.15(0.13) | 3.01(0.12) |
| rh_parahippocampal | | 2.98(0.22) | 3.01(0.22) | 2.97(0.21) |
| rh_paracentral | | 2.71(0.14) | 2.73(0.13) | 2.6(0.13) |
| rh_parsopercularis | | 2.89(0.13) | 2.9(0.13) | 2.75(0.13) |
| rh_parsorbitalis | | 2.98(0.17) | 3.01(0.16) | 2.86(0.16) |
| rh_parstriangularis | | 2.76(0.13) | 2.78(0.13) | 2.61(0.12) |
| rh_pericalcarine | | 1.84(0.13) | 1.85(0.14) | 1.84(0.13) |
| rh_postcentral | | 2.31(0.12) | 2.33(0.12) | 2.22(0.1) |
| rh_posteriorcingulate | | 2.71(0.15) | 2.73(0.14) | 2.59(0.13) |
| rh_precentral | | 2.78(0.11) | 2.81(0.1) | 2.7(0.1) |
| rh_precuneus | | 2.69(0.11) | 2.7(0.11) | 2.56(0.1) |
| rh_rostralanteriorcingulate | | 3.15(0.21) | 3.16(0.21) | 3.04(0.21) |
| rh_rostralmiddlefrontal | | 2.59(0.11) | 2.6(0.11) | 2.43(0.11) |
| rh_superiorfrontal | | 3(0.12) | 3.02(0.11) | 2.83(0.11) |
| rh_superiorparietal | | 2.44(0.11) | 2.46(0.11) | 2.32(0.1) |
| rh_superiortemporal | | 3.12(0.13) | 3.14(0.13) | 3.02(0.12) |
| rh_supramarginal | | 2.85(0.12) | 2.87(0.11) | 2.7(0.11) |
| rh_frontalpole | | 3.03(0.26) | 3.04(0.27) | 2.88(0.25) |
| rh_temporalpole | | 3.73(0.33) | 3.81(0.29) | 3.8(0.28) |
| rh_transversetemporal | | 2.81(0.19) | 2.83(0.19) | 2.74(0.17) |
| rh_insula | | 3.28(0.13) | 3.3(0.13) | 3.18(0.13) |
| **Cortical Surface Area** | | | |  |
| lh_bankssts | | 1087.33(175.35) | 1086.07(173.95) | 1045.18(165.05) |
| lh_caudalanteriorcingulate | | 659.19(133.87) | 652.38(129.8) | 645.8(128.37) |
| lh_caudalmiddlefrontal | | 2409.71(354.94) | 2393.42(350.93) | 2312.23(341.06) |
| lh_cuneus | | 1574.05(216.97) | 1556.29(204.13) | 1522.56(200.13) |
| lh_entorhinal | | 454.4(99.25) | 452.65(95.22) | 448.22(96.28) |
| lh_fusiform | | 3291.28(371.24) | 3258.49(350.93) | 3201.67(343.98) |
| lh_inferiorparietal | | 4830.73(666.54) | 4797.53(639.09) | 4644.57(617.26) |
| lh_inferiortemporal | | 3594.28(471.05) | 3580.77(448.08) | 3539.12(446.04) |
| lh_isthmuscingulate | | 1075.3(157.79) | 1063.75(154.77) | 1038.99(150.55) |
| lh_lateraloccipital | | 5343.16(640.31) | 5268.02(590.03) | 5146.49(584.87) |
| lh_lateralorbitofrontal | | 2836.49(295.66) | 2826.28(282.45) | 2773.75(271.66) |
| lh_lingual | | 3139.56(400.09) | 3107.54(377.97) | 3048.88(377.49) |
| lh_medialorbitofrontal | | 1954.28(252.01) | 1946.58(240.3) | 1898.44(232.6) |
| lh_middletemporal | | 3449.24(433.77) | 3424.72(412.13) | 3383.57(413.46) |
| lh_parahippocampal | | 683.72(76.5) | 677.53(74.45) | 669.9(73.86) |
| lh_paracentral | | 1424.82(164.15) | 1410.26(157.14) | 1377.62(155.68) |
| lh_parsopercularis | | 1742.05(260.66) | 1727.09(258.15) | 1690.67(253.6) |
| lh_parsorbitalis | | 760.3(93.2) | 755.14(90.05) | 735.22(88.96) |
| lh_parstriangularis | | 1449.32(210.8) | 1434.14(207.55) | 1397.24(204) |
| lh_pericalcarine | | 1460.86(250.79) | 1445.22(239.97) | 1435.2(236.24) |
| lh_postcentral | | 4289.79(469.29) | 4230.84(448.48) | 4124.56(439.11) |
| lh_posteriorcingulate | | 1267.4(171.75) | 1251.91(161.63) | 1216.85(158.56) |
| lh_precentral | | 4972.4(510.47) | 4902.51(481.48) | 4814.49(472.84) |
| lh_precuneus | | 4050.05(470.46) | 4012.51(460.18) | 3924.88(450.41) |
| lh_rostralanteriorcingulate | | 860.06(166.07) | 851.25(157.36) | 849.08(155.49) |
| lh_rostralmiddlefrontal | | 6172.35(827.77) | 6099.4(781.94) | 5936.78(746.16) |
| lh_superiorfrontal | | 7806.92(875.08) | 7710.59(819.78) | 7574.41(815.87) |
| lh_superiorparietal | | 5700.75(650.03) | 5649.73(642.41) | 5512.71(622.02) |
| lh_superiortemporal | | 4159.31(486.53) | 4117.74(458.43) | 4052.87(456.74) |
| lh_supramarginal | | 4362.91(682.94) | 4296.79(637.34) | 4175.76(617.61) |
| lh_frontalpole | | 287.79(35.43) | 287.65(33.03) | 279.23(33.45) |
| lh_temporalpole | | 497.12(61.15) | 492.35(62.04) | 490.17(60.17) |
| lh_transversetemporal | | 457.93(71.96) | 452.75(67.75) | 441.03(65.29) |
| lh_insula | | 2418.71(247.01) | 2395.92(241.65) | 2339.71(233.63) |
| rh_bankssts | | 971.94(138.41) | 968.29(133.87) | 928.93(126.24) |
| rh_caudalanteriorcingulate | | 746.64(149.81) | 740.06(145.36) | 732.31(143.89) |
| rh_caudalmiddlefrontal | | 2284.85(345.8) | 2273.25(332.72) | 2199(323.5) |
| rh_cuneus | | 1671.57(221.43) | 1649.81(208.68) | 1618.28(205.96) |
| rh_entorhinal | | 403.28(81.22) | 395.06(76.18) | 392.18(76.34) |
| rh_fusiform | | 3179.58(378.69) | 3146.57(361.52) | 3094.5(353.96) |
| rh_inferiorparietal | | 5727.84(787.26) | 5672.69(747.64) | 5499.26(725.02) |
| rh_inferiortemporal | | 3457.63(453.71) | 3429.77(423.03) | 3365.27(416.11) |
| rh_isthmuscingulate | | 974.88(137.49) | 963.36(136.74) | 940.36(133.53) |
| rh_lateraloccipital | | 5313.62(675.61) | 5242.52(639.93) | 5129.45(629.23) |
| rh_lateralorbitofrontal | | 2753.51(314.77) | 2738.88(303.4) | 2691.19(295.16) |
| rh_lingual | | 3282.53(432.97) | 3244.16(407.25) | 3188.51(409.39) |
| rh_medialorbitofrontal | | 2038.84(229.94) | 2029.44(214.41) | 1985.97(207.12) |
| rh_middletemporal | | 3798.17(446.61) | 3767.4(416.92) | 3699.65(417.65) |
| rh_parahippocampal | | 649.64(75.34) | 642.4(72.94) | 634.49(72.27) |
| rh_paracentral | | 1574.85(184.2) | 1560.83(177.82) | 1527.22(175.44) |
| rh_parsopercularis | | 1468.42(202.85) | 1457.08(202.33) | 1423.61(195.71) |
| rh_parsorbitalis | | 902.91(113.84) | 895.45(107.94) | 871.37(106.5) |
| rh_parstriangularis | | 1655.98(246.3) | 1640.16(245.11) | 1599.98(242.66) |
| rh_pericalcarine | | 1618.34(268.4) | 1597.56(252.22) | 1588.67(250.2) |
| rh_postcentral | | 4156.93(459.47) | 4108.55(427.8) | 3995.77(414.39) |
| rh_posteriorcingulate | | 1280.93(174.47) | 1270.85(167.84) | 1235.14(164.46) |
| rh_precentral | | 4968.74(517.72) | 4908.88(490.97) | 4817.31(482.13) |
| rh_precuneus | | 4221.02(495.81) | 4186.55(488.24) | 4099.09(476.73) |
| rh_rostralanteriorcingulate | | 639.63(123.72) | 636.14(123.54) | 633.15(123.01) |
| rh_rostralmiddlefrontal | | 6324.34(868.08) | 6238.81(807.42) | 6068.98(765.84) |
| rh_superiorfrontal | | 7522.45(875.21) | 7417.59(823.54) | 7283.36(815.93) |
| rh_superiorparietal | | 5637.44(609.52) | 5581.44(590.11) | 5439.05(574.07) |
| rh_superiortemporal | | 3877.63(411.32) | 3829.01(382.81) | 3773.98(383.33) |
| rh_supramarginal | | 3918.42(530.59) | 3877.85(510.56) | 3761.9(493.03) |
| rh_frontalpole | | 344.92(42.64) | 343.16(41.29) | 332.65(41.62) |
| rh_temporalpole | | 478.59(61.73) | 475.85(62.15) | 471.42(60.05) |
| rh_transversetemporal | | 344.61(47.44) | 339.01(44.98) | 330.82(43.54) |
| rh_insula | | 2359.93(257.1) | 2332.86(250.44) | 2285.51(240.11) |
| **Subcortical Volumes** | | | |  |
| Left.Lateral.Ventricle | 5438.56(2468.86) | | 5377.41(2352.85) | 5842.96(2573.51) |
| Left.Cerebellum.Cortex | 56407.88(5429.48) | | 56089.24(5300.28) | 54724.97(5338.38) |
| Left.Thalamus.Proper | 7574.3(690.47) | | 7552.02(681.69) | 7417.59(702.59) |
| Left.Caudate | 4148.2(470.15) | | 4133.28(454.65) | 4016.63(446.68) |
| Left.Putamen | 5809.54(595.43) | | 5766.6(601.88) | 5591.23(605.1) |
| Left.Pallidum | 1962.27(221.66) | | 1953.07(220.77) | 2017.7(236.77) |
| Left.Hippocampus | 4118.04(365.47) | | 4100.27(353.29) | 4105.31(366.2) |
| Left.Amygdala | 1740.13(209.26) | | 1733.71(201.38) | 1759.82(204.77) |
| Left.Accumbens.area | 710.65(111.26) | | 713.03(106.65) | 701.25(111.54) |
| Left.VentralDC | 4076.3(378.46) | | 4057.66(366.95) | 4085.53(393.3) |
| Right.Lateral.Ventricle | 5008.99(2335.59) | | 5000.02(2307.8) | 5456.96(2523.33) |
| Right.Cerebellum.Cortex | 56725.56 (5499.76) | | 56360.06(5391.72) | 54977.23(5436.14) |
| Right.Thalamus.Proper | 7392.66(674.44) | | 7377.42(668.76) | 7258.5(692) |
| Right.Caudate | 4233.95(481.43) | | 4210.6(459.82) | 4097.22(456.36) |
| Right.Putamen | 5823.66(587.28) | | 5765.27(588.68) | 5613.67(587.34) |
| Right.Pallidum | 1867.51(203.37) | | 1849.63(199.6) | 1891.6(210.36) |
| Right.Hippocampus | 4250.92(387.82) | | 4223.82(373.88) | 4217.32(386.86) |
| Right.Amygdala | 1924.9(230.17) | | 1911.25(221.68) | 1917.6(234.95) |
| Right.Accumbens.area | 773.37(107.42) | | 767.85(104.19) | 761.06(106.28) |
| Right.VentralDC | 4107.45(368.12) | | 4094.63(360.59) | 4112.71(391.1) |
| ^1^Denotes differences between the total sample and the development change sample at baseline at p<0.05 uncorrected; ^2^ Denotes differences in the development change sample between their baseline and follow-up assessments at p<0.05 uncorrected | | | | |

1. **Sparse Canonical Correlation Analyses: Cortical Thickness**

**
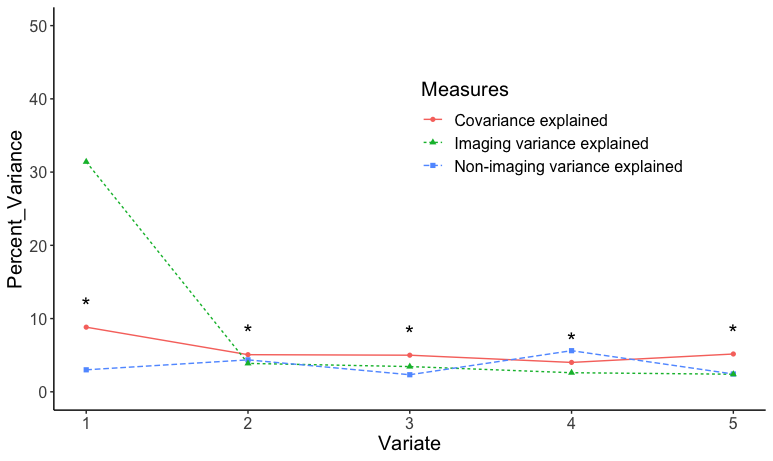
Supplemental Figure S2. Cortical Thickness at baseline: covariance and variance explained by the canonical variates.** Five canonical modes were statistically significant at an FDR-corrected P value<0.001 (*). We reported only the first canonical mode because 1. In testing the parameters of the trained model on the test set only the first correlation in the test set was at least >80% of that of the training set 2. Only the first mode had a Median RR-Score of >0.80

**Supplemental Figure S3. Cortical Thickness developmental change: covariance and variance explained by the canonical variates.** Two canonical modes were statistically significant at an FDR-corrected P value<0.001 (*). We reported only the first canonical mode because 1. In testing the parameters of the trained model on the test set only the correlation between the first pair of variates in the test set was at least >80% of that of the training set 2. Only the first mode had a Median RR-Score of >0.80

**
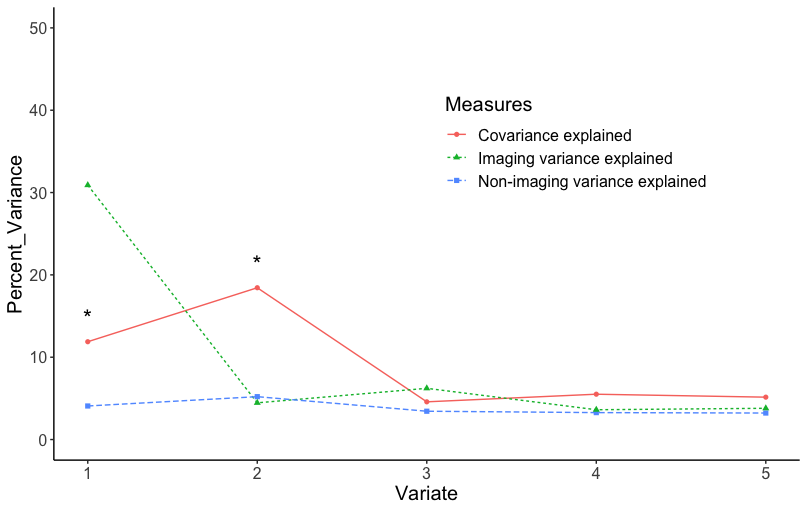
**

| **Table S6. Cortical Thickness at baseline: Canonical weights (w) of the non-imaging variables; only non-zero weights are shown** | |
| --- | --- |
| **Variables with \|w\|>0.1** | |
| Sex | 0.91 |
| LEQ Sexuality | -0.12 |
| LEQ Family | -0.12 |
| Age | -0.38 |
| **Variables with \|w\|<0.1** | |
| SURPS Impulsivity | -0.02 |
| Number of Negative Life Events | -0.03 |
| ESPAD Frequency of Lifetime Smoking | -0.04 |
| Body Mass Index | -0.05 |
| Pubertal Stage | -0.05 |
| ESPAD= European School Survey Project on Alcohol and Other Drugs; LEQ=Life Event Questionnaire; SURPS=Substance Use Risk Profile Scale; NEO=NEO-Five Factor Personality Inventory’ | |

| **Table S7. Cortical Thickness at baseline: Canonical loadings (ρ) of the non-imaging variables** | |
| --- | --- |
| **Variables with ρ > \|0.1\|** | |
| Sex | 0.24 |
| LEQ Family | -0.1 |
| LEQ Sexuality | -0.1 |
| Age | -0.14 |
| **Variables with ρ< \|0.1\|** | |
| Maternal Prenatal Alcohol Use | 0.06 |
| Child Experience Affirmation | 0.05 |
| Paternal Education | 0.05 |
| Intelligence g-Factor | 0.05 |
| Family Stressors Work/Pressure | 0.04 |
| Average Grade | 0.03 |
| Maternal Education | 0.04 |
| Family Stressors Illness | 0.03 |
| Family History of Psychiatric Disorders | 0.03 |
| Child Experience Discipline | 0.02 |
| Living with Both Parents | 0.01 |
| Maternal Prenatal Medical Illness | 0.01 |
| Pregnancy Complications | 0.01 |
| Breastfed | 0.01 |
| NEO Agreeableness | 0.01 |
| SURPS Sensation Seeking | 0.01 |
| Height | 0 |
| Family Stressors Relationship/Addiction | -0.01 |
| Child Experience Rules | -0.01 |
| Social Aptitude Scale | -0.01 |
| Paternal Prenatal Smoking | -0.01 |
| ESPAD Frequency of Lifetime Alcohol Use | -0.01 |
| Birth Weight | -0.02 |
| ESPAD Frequency of Cannabis Use in the Preceding Month | -0.02 |
| Perpetrator of Bullying | -0.02 |
| LEQ Relocation | -0.02 |
| NEO Conscientiousness | -0.02 |
| ESPAD Frequency of Smoking in the Preceding 30 Days | -0.03 |
| ESPAD Frequency of Alcohol Use in the Preceding Month | -0.03 |
| Victim of Bullying | -0.03 |
| NEO Neuroticism | -0.03 |
| SURPS Hopelessness | -0.03 |
| Family Stressors Socioeconomic/Housing | -0.04 |
| ESPAD Frequency of Lifetime Cannabis use | -0.04 |
| Truancy | -0.04 |
| LEQ Autonomy | -0.04 |
| LEQ Deviance | -0.04 |
| SURPS Anxiety Sensitivity | -0.04 |
| TCI Novelty Seeking | -0.04 |
| Maternal Prenatal Smoking | -0.05 |
| LEQ Accident | -0.05 |
| LEQ Others | -0.05 |
| LEQ Distress | -0.06 |
| Weight | -0.06 |
| NEO Openness | -0.06 |
| Psychiatric Diagnosis | -0.07 |
| NEO Extroversion | -0.07 |
| ESPAD Frequency of ESPAD Frequency of Lifetime Smoking | -0.08 |
| Number of Negative Life Events | -0.08 |
| Body Mass Index | -0.08 |
| Pubertal Stage | -0.08 |
| SURPS Impulsivity | -0.08 |
| ESPAD= European School Survey Project on Alcohol and Other Drugs; LEQ=Life Event Questionnaire; NEO= NEO-Five Factor Personality Inventory; SURPS= Substance Use Risk Profile Scale TCI=Temperament and Character Inventory | |

| **Table S8. Cortical Thickness at baseline: Canonical weights (w) of the imaging variables; only non-zero weights are shown** | |
| --- | --- |
| **Variables with \|w\|>0.1** | |
| lh_rostralmiddlefrontal | 0.4 |
| lh_MeanThickness | 0.31 |
| rh_MeanThickness | 0.31 |
| rh_insula | 0.28 |
| lh_lateralorbitofrontal | 0.26 |
| rh_rostralmiddlefrontal | 0.26 |
| lh_precuneus | 0.24 |
| lh_superiorparietal | 0.24 |
| rh_inferiorparietal | 0.24 |
| lh_inferiorparietal | 0.23 |
| lh_parsopercularis | 0.22 |
| lh_insula | 0.18 |
| rh_superiorparietal | 0.18 |
| lh_lateraloccipital | 0.17 |
| rh_precuneus | 0.17 |
| rh_lateraloccipital | 0.11 |
| lh_medialorbitofrontal | 0.1 |
| **Variables with \|w\|<0.1** | |
| rh_cuneus | 0.08 |
| lh_cuneus | 0.06 |
| rh_caudalmiddlefrontal | 0.06 |
| rh_middletemporal | 0.04 |
| rh_parsopercularis | 0.04 |
| rh_supramarginal | 0.04 |
| rh_superiorfrontal | 0.01 |

| **Table S9. Cortical Thickness at baseline: Canonical loadings (ρ) of the imaging variables** | |
| --- | --- |
| **Variables with \|ρ\|> 0.1** | |
| lh_rostralmiddlefrontal | 0.26 |
| lh_MeanThickness | 0.23 |
| rh_insula | 0.23 |
| rh_MeanThickness | 0.23 |
| lh_lateralorbitofrontal | 0.22 |
| lh_superiorparietal | 0.22 |
| rh_inferiorparietal | 0.22 |
| rh_rostralmiddlefrontal | 0.22 |
| lh_inferiorparietal | 0.21 |
| lh_parsopercularis | 0.21 |
| lh_precuneus | 0.21 |
| lh_lateraloccipital | 0.2 |
| lh_insula | 0.2 |
| rh_precuneus | 0.2 |
| rh_superiorparietal | 0.2 |
| lh_medialorbitofrontal | 0.18 |
| rh_lateraloccipital | 0.18 |
| lh_cuneus | 0.17 |
| rh_caudalmiddlefrontal | 0.17 |
| rh_cuneus | 0.17 |
| rh_middletemporal | 0.16 |
| rh_parsopercularis | 0.16 |
| rh_superiorfrontal | 0.16 |
| rh_supramarginal | 0.16 |
| lh_lingual | 0.15 |
| lh_superiorfrontal | 0.15 |
| rh_bankssts | 0.15 |
| rh_inferiortemporal | 0.15 |
| rh_lateralorbitofrontal | 0.15 |
| rh_superiortemporal | 0.15 |
| lh_caudalmiddlefrontal | 0.14 |
| lh_paracentral | 0.14 |
| lh_parstriangularis | 0.14 |
| lh_precentral | 0.14 |
| rh_lingual | 0.14 |
| rh_medialorbitofrontal | 0.14 |
| lh_inferiortemporal | 0.13 |
| rh_fusiform | 0.13 |
| lh_postcentral | 0.12 |
| lh_supramarginal | 0.12 |
| rh_precentral | 0.12 |
| rh_paracentral | 0.11 |
| rh_postcentral | 0.11 |
| lh_middletemporal | 0.1 |
| lh_superiortemporal | 0.1 |
| rh_parstriangularis | 0.1 |
| **Variables with \|ρ\|< 0.1** | |
| lh_fusiform | 0.09 |
| lh_entorhinal | 0.08 |
| lh_parsorbitalis | 0.08 |
| lh_bankssts | 0.07 |
| rh_pericalcarine | 0.06 |
| lh_pericalcarine | 0.05 |
| lh_frontalpole | 0.05 |
| lh_temporalpole | 0.04 |
| rh_parsorbitalis | 0.04 |
| rh_frontalpole | 0.04 |
| rh_posteriorcingulate | 0.03 |
| rh_rostralanteriorcingulate | 0.03 |
| rh_temporalpole | 0.03 |
| rh_transversetemporal | 0.03 |
| lh_isthmuscingulate | 0.01 |
| lh_posteriorcingulate | 0.01 |
| rh_entorhinal | 0.01 |
| rh_isthmuscingulate | 0.01 |
| rh_caudalanteriorcingulate | -0.04 |
| lh_caudalanteriorcingulate | -0.05 |
| lh_parahippocampal | -0.05 |
| lh_rostralanteriorcingulate | -0.05 |
| rh_parahippocampal | -0.06 |
| lh_transversetemporal | -0.07 |

| **Table S10. Cortical thickness developmental change: Canonical weights (w) of the non-imaging variables; only non-zero weights are shown** | |
| --- | --- |
| **Variables with \|w\|> 0.1** | |
| Height | -0.15 |
| ESPAD Frequency of Alcohol Use in the Preceding Month | -0.33 |
| Age | -0.93 |
| **Variables with \|w\|< 0.1** | |
| ESPAD Frequency of Lifetime Alcohol Use | -0.04 |
| ESPAD Frequency of Lifetime Cannabis use | -0.05 |
| Perpetrator of Bullying | -0.05 |
| ESPAD Frequency of Cannabis Use in the Preceding Month | -0.09 |
| ESPAD= European School Survey Project on Alcohol and Other Drugs | |

| **Table S11. Cortical thickness for developmental change: Canonical loadings (ρ) of the non-imaging variables** | |
| --- | --- |
| **Variables with \|ρ\|> 0.1** | |
| SURPS Sensation Seeking | -0.1 |
| ESPAD Frequency of Lifetime Alcohol Use | -0.11 |
| ESPAD Frequency of Lifetime Cannabis use | -0.11 |
| Perpetrator of Bullying | -0.11 |
| ESPAD Frequency of Cannabis Use in the Preceding Month | -0.12 |
| Height | -0.13 |
| ESPAD Frequency of Alcohol Use in the Preceding Month | -0.17 |
| Age | -0.3 |
| **Variables with \|ρ\|< 0.1** | |
| Average Grade | 0.08 |
| Body Mass Index | 0.08 |
| LEQ Distress | 0.05 |
| Family Stressors Illness | 0.04 |
| NEO Agreeableness | 0.04 |
| SURPS Anxiety Sensitivity | 0.04 |
| Truancy | 0.03 |
| LEQ Family | 0.03 |
| LEQ Relocation | 0.03 |
| Family Stressors Socioeconomic/Housing | 0.01 |
| Number of Negative Life Events | 0.01 |
| Child Experience Affirmation | 0 |
| LEQ Autonomy | 0 |
| Weight | 0 |
| Child Experience Rules | -0.01 |
| NEO Neuroticism | -0.01 |
| Sex | -0.02 |
| Living with Both Parents | -0.02 |
| Family Stressors Work/Pressure | -0.02 |
| Social Aptitude Scale | -0.02 |
| LEQ Accident | -0.02 |
| Child Experience Discipline | -0.03 |
| NEO Extroversion | -0.03 |
| NEO Conscientiousness | -0.03 |
| Family Stressors Relationship/Addiction | -0.04 |
| ESPAD Frequency of Smoking in the Preceding Month | -0.04 |
| TCI Novelty Seeking | -0.04 |
| Psychiatric Diagnosis | -0.05 |
| LEQ Sexuality | -0.05 |
| SURPS Hopelessness | -0.05 |
| Victim of Bullying | -0.06 |
| LEQ Others | -0.06 |
| ESPAD Frequency of Lifetime Smoking | -0.07 |
| LEQ Deviance | -0.07 |
| NEO Openness | -0.08 |
| SURPS Impulsivity | -0.08 |
| ESPAD= European School Survey Project on Alcohol and Other Drugs; LEQ=Life Event Questionnaire; NEO= NEO-Five Factor Personality Inventory; SURPS= Substance Use Risk Profile Scale; TCI=Temperament and Character Inventory | |

| **Table S12. Cortical Thickness for developmental change: Canonical weights (w) of the imaging variables; only non-zero weights are shown** | |
| --- | --- |
| **Variables with \|w\|>0.1** | |
| lh_superiorfrontal | 0.29 |
| lh_supramarginal | 0.26 |
| lh_bankssts | 0.25 |
| rh_parsopercularis | 0.25 |
| rh_superiorfrontal | 0.25 |
| rh_posteriorcingulate | 0.24 |
| lh_parstriangularis | 0.23 |
| lh_rostralmiddlefrontal | 0.22 |
| rh_parstriangularis | 0.21 |
| lh_parsopercularis | 0.2 |
| lh_caudalmiddlefrontal | 0.19 |
| rh_medialorbitofrontal | 0.19 |
| rh_rostralmiddlefrontal | 0.19 |
| lh_superiortemporal | 0.18 |
| lh_inferiorparietal | 0.17 |
| lh_parsorbitalis | 0.17 |
| rh_rostralanteriorcingulate | 0.14 |
| lh_middletemporal | 0.13 |
| rh_caudalmiddlefrontal | 0.13 |
| rh_parsorbitalis | 0.13 |
| lh_caudalanteriorcingulate | 0.12 |
| rh_superiortemporal | 0.12 |
| lh_lateralorbitofrontal | 0.11 |
| rh_lateralorbitofrontal | 0.11 |
| lh_rostralanteriorcingulate | 0.1 |
| lh_frontalpole | 0.1 |
| **Variables with \|w\|<0.1** | |
| rh_caudalanteriorcingulate | 0.09 |
| rh_isthmuscingulate | 0.09 |
| rh_precuneus | 0.09 |
| lh_lateraloccipital | 0.08 |
| lh_precuneus | 0.08 |
| lh_superiorparietal | 0.08 |
| rh_inferiorparietal | 0.08 |
| lh_posteriorcingulate | 0.07 |
| rh_supramarginal | 0.07 |
| lh_precentral | 0.06 |
| rh_middletemporal | 0.06 |
| lh_insula | 0.05 |
| rh_bankssts | 0.05 |
| rh_inferiortemporal | 0.04 |
| rh_fusiform | 0.03 |
| rh_lateraloccipital | 0.03 |
| rh_insula | 0.03 |
| lh_medialorbitofrontal | 0.01 |

| **Table S13.** **Cortical thickness developmental change: Canonical loadings (ρ) of the variables** | |
| --- | --- |
| **Variables with \|ρ\|> 0.1** | |
| lh_superiorfrontal | 0.28 |
| lh_bankssts | 0.26 |
| lh_supramarginal | 0.26 |
| rh_parsopercularis | 0.26 |
| rh_posteriorcingulate | 0.26 |
| rh_superiorfrontal | 0.26 |
| lh_parstriangularis | 0.25 |
| lh_rostralmiddlefrontal | 0.25 |
| lh_parsopercularis | 0.24 |
| rh_parstriangularis | 0.24 |
| lh_caudalmiddlefrontal | 0.23 |
| lh_superiortemporal | 0.23 |
| rh_medialorbitofrontal | 0.23 |
| rh_rostralmiddlefrontal | 0.23 |
| lh_inferiorparietal | 0.22 |
| lh_parsorbitalis | 0.22 |
| rh_rostralanteriorcingulate | 0.21 |
| lh_middletemporal | 0.2 |
| rh_caudalmiddlefrontal | 0.2 |
| rh_parsorbitalis | 0.2 |
| rh_superiortemporal | 0.2 |
| lh_caudalanteriorcingulate | 0.19 |
| lh_lateralorbitofrontal | 0.19 |
| lh_rostralanteriorcingulate | 0.19 |
| lh_frontalpole | 0.19 |
| rh_lateralorbitofrontal | 0.19 |
| lh_lateraloccipital | 0.18 |
| lh_precuneus | 0.18 |
| lh_superiorparietal | 0.18 |
| rh_caudalanteriorcingulate | 0.18 |
| rh_inferiorparietal | 0.18 |
| rh_isthmuscingulate | 0.18 |
| rh_precuneus | 0.18 |
| lh_posteriorcingulate | 0.17 |
| lh_precentral | 0.17 |
| rh_middletemporal | 0.17 |
| rh_supramarginal | 0.17 |
| lh_insula | 0.16 |
| rh_bankssts | 0.16 |
| rh_inferiortemporal | 0.16 |
| rh_fusiform | 0.15 |
| rh_lateraloccipital | 0.15 |
| rh_insula | 0.15 |
| lh_medialorbitofrontal | 0.14 |
| rh_superiorparietal | 0.14 |
| lh_isthmuscingulate | 0.13 |
| lh_postcentral | 0.13 |
| rh_paracentral | 0.13 |
| rh_precentral | 0.13 |
| rh_frontalpole | 0.13 |
| rh_transversetemporal | 0.13 |
| lh_paracentral | 0.12 |
| rh_postcentral | 0.12 |
| lh_transversetemporal | 0.11 |
| lh_cuneus | 0.1 |
| lh_inferiortemporal | 0.1 |
| rh_cuneus | 0.1 |
| **Variables with \|ρ\|< 0.1** | |
| lh_fusiform | 0.09 |
| lh_pericalcarine | 0.08 |
| rh_lingual | 0.07 |
| lh_parahippocampal | 0.06 |
| rh_parahippocampal | 0.03 |
| lh_lingual | 0.02 |
| lh_temporalpole | 0.02 |
| rh_pericalcarine | 0.01 |
| rh_entorhinal | 0 |
| rh_temporalpole | -0.01 |
| lh_entorhinal | -0.06 |

**3. Sparse Canonical Correlation Analyses: Cortical Surface Area**

**
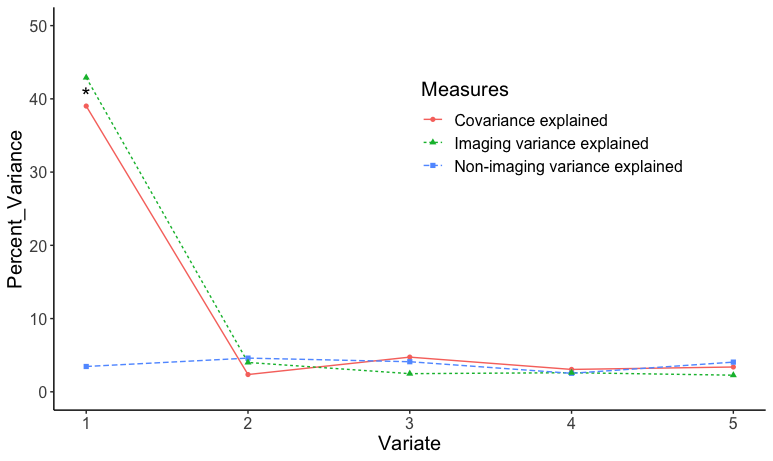
Supplemental Figure S4. Cortical Surface Area at baseline: covariance and variance explained by the canonical variates.** One canonical mode were statistically significant at an FDR-corrected P value<0.001 (*). Therefore, we reported only the first canonical mode. In addition 1. In testing the parameters of the trained model on the test set only the correlation between the first pair of variates in the test set was at least >80% of that of the training set 2. Only the first mode had a Median RR-Score of >0.80

**Supplemental Figure S5. Cortical Surface Area developmental change: covariance and variance explained by the canonical variates.** Two canonical modes were statistically significant at an FDR-corrected P value<0.001(*). We reported only the first canonical mode because: 1. In testing the parameters of the trained model on the test set only the correlation between the first pair of variates in the test set was at least >80% of that of the training set 2. Only the first mode had a Median RR-Score of >0.80
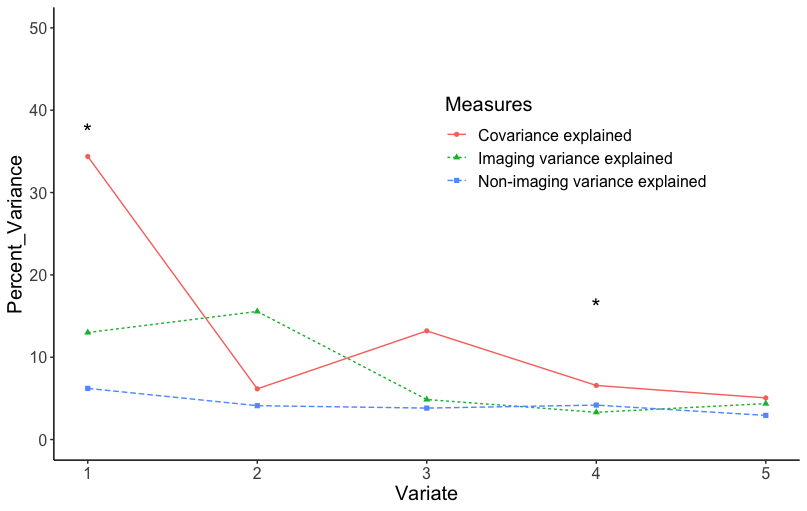


| **Table S14.Cortical Surface Area at baseline: Canonical weights (w) of the non-imaging variables; only non-zero weights are shown** |
| --- |

| **Variables with \|w\|>0.1** |
| --- |

| Sex | 0.89 |
| --- | --- |
| Height | 0.38 |
| Intelligence g-Factor | 0.2 |
| Birth Weight | 0.11 |
| NEO Neuroticism | -0.13 |

| **Variables with \|w\|<0.1** |
| --- |

| Weight | 0.02 |
| --- | --- |
| Paternal Education | 0.01 |
| NEO=NEO-Five Factor Personality Inventory | |

| **Table S15. Cortical Surface Area at baseline: Canonical loadings (ρ) of the non-imaging variables to the imaging variate** | |
| --- | --- |
| **Variables \|ρ\| > 0.1** | |
| Sex | 0.58 |
| Height | 0.33 |
| Intelligence g-Factor | 0.23 |
| Birth Weight | 0.19 |
| Paternal Education | 0.14 |
| Maternal Education | 0.12 |
| Weight | 0.12 |
| SURPS Sensation Seeking | 0.1 |
| Paternal Prenatal Smoking | -0.11 |
| LEQ Distress | -0.11 |
| SURPS Anxiety Sensitivity | -0.12 |
| Maternal Prenatal Smoking | -0.14 |
| NEO Neuroticism | -0.2 |
| **Variables \|ρ\| < 0.1** | |
| Perpetrator of Bullying | 0.08 |
| LEQ Deviance | 0.08 |
| Breastfed | 0.07 |
| Average Grade | 0.06 |
| LEQ Autonomy | 0.06 |
| Family Stressors Work/Pressure | 0.04 |
| Maternal Prenatal Alcohol Use | 0.04 |
| LEQ Relocation | 0.03 |
| Child Experience Affirmation | 0.02 |
| Family History of Psychiatric Disorders | 0.02 |
| ESPAD Frequency of Lifetime Cannabis use | 0.02 |
| Living with Both Parents | 0.01 |
| Child Experience Discipline | 0.01 |
| Social Aptitude Scale | 0.01 |
| Pregnancy Complications | 0.01 |
| Pubertal Stage | 0.01 |
| Child Experience Rules | 0 |
| ESPAD Frequency of Cannabis Use in the Preceding Month | 0 |
| Victim of Bullying | -0.01 |
| NEO Conscientiousness | -0.01 |
| LEQ Accident | -0.02 |
| LEQ Others | -0.02 |
| Family Stressors Relationship/Addiction | -0.03 |
| NEO Extroversion | -0.03 |
| NEO Openness | -0.03 |
| NEO Agreeableness | -0.03 |
| ESPAD Frequency of Lifetime Alcohol Use | -0.04 |
| ESPAD Frequency of Alcohol Use in the Preceding Month | -0.04 |
| SURPS Hopelessness | -0.04 |
| TCI Novelty Seeking | -0.04 |
| Family Stressors Illness | -0.05 |
| Number of Negative Life Events | -0.05 |
| SURPS Impulsivity | -0.05 |
| ESPAD Frequency of Smoking in the Preceding 30 Days | -0.06 |
| Body Mass Index | -0.06 |
| Age | -0.07 |
| Maternal Prenatal Medical Illness | -0.07 |
| Truancy | -0.07 |
| Psychiatric Diagnosis | -0.08 |
| LEQ Family | -0.08 |
| LEQ Sexuality | -0.08 |
| Family Stressors Socioeconomic/Housing | -0.09 |
| ESPAD Frequency of ESPAD Frequency of Lifetime Smoking | -0.09 |
| LEQ=Life Event Questionnaire; NEO= NEO-Five Factor Personality Inventory; SURPS= Substance Use Risk Profile Scale; TCI=Temperament and Character Inventory | |

| **Table S16. Cortical Surface Area at baseline: Canonical weights (w) of the imaging variables; only non-zero weights are shown** | |
| --- | --- |
| **Variables with \|w\|>0.1** | |
| Total Surface Area | 0.46 |
| lh_rostralmiddlefrontal | 0.25 |
| lh_superiortemporal | 0.24 |
| rh_fusiform | 0.23 |
| rh_insula | 0.23 |
| rh_lateraloccipital | 0.21 |
| lh_precentral | 0.2 |
| lh_superiorfrontal | 0.2 |
| lh_insula | 0.2 |
| rh_inferiortemporal | 0.2 |
| rh_rostralmiddlefrontal | 0.18 |
| rh_superiorfrontal | 0.18 |
| lh_supramarginal | 0.17 |
| rh_precentral | 0.17 |
| lh_fusiform | 0.15 |
| rh_inferiorparietal | 0.15 |
| rh_medialorbitofrontal | 0.15 |
| rh_middletemporal | 0.15 |
| lh_lateraloccipital | 0.12 |
| lh_parsorbitalis | 0.12 |
| lh_postcentral | 0.12 |
| lh_precuneus | 0.12 |
| rh_precuneus | 0.12 |
| lh_inferiortemporal | 0.11 |
| lh_middletemporal | 0.11 |
| lh_lateralorbitofrontal | 0.1 |
| rh_postcentral | 0.1 |
| **Variables with \|w\|<0.1** | |
| rh_lateralorbitofrontal | 0.09 |
| rh_parsorbitalis | 0.07 |
| rh_superiortemporal | 0.05 |
| rh_isthmuscingulate | 0.03 |

| **Table S17. Cortical Surface Area at baseline: Canonical loadings (ρ) of the imaging variables to the non-imaging variate** | |
| --- | --- |
| **Variables \|ρ\| > 0.1** | |
| Total Surface Area | 0.6 |
| lh_rostralmiddlefrontal | 0.51 |
| lh_superiortemporal | 0.51 |
| rh_fusiform | 0.5 |
| rh_insula | 0.5 |
| lh_precentral | 0.49 |
| lh_superiorfrontal | 0.49 |
| lh_insula | 0.49 |
| rh_inferiortemporal | 0.49 |
| rh_lateraloccipital | 0.49 |
| lh_supramarginal | 0.48 |
| rh_precentral | 0.48 |
| rh_rostralmiddlefrontal | 0.48 |
| rh_superiorfrontal | 0.48 |
| lh_fusiform | 0.47 |
| rh_medialorbitofrontal | 0.47 |
| lh_parsorbitalis | 0.46 |
| rh_inferiorparietal | 0.46 |
| rh_middletemporal | 0.46 |
| lh_inferiortemporal | 0.45 |
| lh_lateraloccipital | 0.45 |
| lh_middletemporal | 0.45 |
| lh_postcentral | 0.45 |
| lh_precuneus | 0.45 |
| rh_precuneus | 0.45 |
| lh_lateralorbitofrontal | 0.44 |
| rh_lateralorbitofrontal | 0.44 |
| rh_postcentral | 0.44 |
| rh_parsorbitalis | 0.43 |
| rh_superiortemporal | 0.42 |
| rh_isthmuscingulate | 0.41 |
| lh_isthmuscingulate | 0.4 |
| lh_medialorbitofrontal | 0.4 |
| rh_parstriangularis | 0.38 |
| rh_posteriorcingulate | 0.38 |
| rh_supramarginal | 0.38 |
| lh_parstriangularis | 0.37 |
| rh_cuneus | 0.37 |
| rh_paracentral | 0.37 |
| rh_superiorparietal | 0.37 |
| lh_posteriorcingulate | 0.36 |
| lh_bankssts | 0.35 |
| lh_cuneus | 0.35 |
| lh_inferiorparietal | 0.35 |
| lh_temporalpole | 0.35 |
| lh_paracentral | 0.34 |
| lh_superiorparietal | 0.34 |
| rh_bankssts | 0.34 |
| lh_rostralanteriorcingulate | 0.33 |
| rh_parahippocampal | 0.33 |
| rh_transversetemporal | 0.33 |
| lh_caudalmiddlefrontal | 0.32 |
| rh_rostralanteriorcingulate | 0.32 |
| lh_lingual | 0.31 |
| rh_caudalmiddlefrontal | 0.31 |
| rh_parsopercularis | 0.31 |
| rh_frontalpole | 0.31 |
| lh_parsopercularis | 0.3 |
| lh_parahippocampal | 0.29 |
| lh_transversetemporal | 0.29 |
| rh_lingual | 0.29 |
| lh_frontalpole | 0.28 |
| rh_temporalpole | 0.28 |
| rh_entorhinal | 0.26 |
| lh_entorhinal | 0.25 |
| rh_caudalanteriorcingulate | 0.22 |
| rh_pericalcarine | 0.22 |
| lh_pericalcarine | 0.21 |
| lh_caudalanteriorcingulate | 0.2 |

| **Table S18. Cortical Surface Area developmental change: Canonical weights (w) of the non- imaging variables; only non-zero weights are shown** | |
| --- | --- |
| **Variables with \|w\|>0.1** | |
| Height | 0.67 |
| Sex | 0.58 |
| Weight | 0.37 |
| Age | 0.24 |
| **Variables with \|w\|<0.1** | |
| ESPAD Frequency of Lifetime Cannabis use | 0.09 |
| LEQ Deviance | 0.05 |
| SURPS Sensation Seeking | 0.03 |
| ESPAD Frequency of Cannabis Use in the Preceding Month | 0.02 |
| NEO Neuroticism | -0.01 |
| LEQ Accident | -0.02 |
| Number of Negative Life Events | -0.04 |
| LEQ Distress | -0.06 |

| ESPAD= European School Survey Project on Alcohol and Other Drugs; LEQ=Life Event Questionnaire; NEO= NEO-Five Factor Personality Inventory; SURPS Substance Use Risk Profile Scale |
| --- |

| **Table S19. Cortical Surface Area developmental change: Canonical loadings (ρ) of the non-imaging variables** | |
| --- | --- |
| **Variables \|ρ\|> 0.1** | |
| Height | 0.54 |
| Sex | 0.48 |
| Weight | 0.34 |
| Age | 0.25 |
| ESPAD Frequency of Lifetime Cannabis use | 0.14 |
| LEQ Deviance | 0.12 |
| ESPAD Frequency of Cannabis Use in the Preceding Month | 0.1 |
| SURPS Sensation Seeking | 0.1 |
| LEQ Accident | -0.1 |
| Number of Negative Life Events | -0.11 |
| LEQ Distress | -0.12 |
| SURPS Anxiety Sensitivity | -0.13 |
| **Variables \|ρ\| < 0.1** | |
| Perpetrator of Bullying | 0.07 |
| ESPAD Frequency of Lifetime Alcohol use | 0.06 |
| SURPS Impulsivity | 0.05 |
| ESPAD Frequency of Smoking in the Preceding Month | 0.05 |
| Body Mass Index | 0.05 |
| TCI Novelty Seeking | 0.04 |
| ESPAD Frequency of Alcohol Use in the Preceding Month | 0.03 |
| ESPAD Frequency of Lifetime Smoking | 0.03 |
| Victim of Bullying | 0.02 |
| SURPS Hopelessness | 0.02 |
| NEO Openness | 0.02 |
| LEQ Relocation | 0.02 |
| Child Experience Discipline | 0.02 |
| LEQ Autonomy | 0.01 |
| Family Stressors Relationship/Addiction | 0.01 |
| Average Grade | 0.01 |
| Family Stressors Illness | 0.01 |
| Family Stressors Work/Pressure | 0 |
| Family Stressors Socioeconomic/Housing | 0 |
| Living with Both Parents | -0.01 |
| LEQ Others | -0.02 |
| NEO Extroversion | -0.03 |
| NEO Agreeableness | -0.03 |
| Child Experience Affirmation | -0.04 |
| Social Aptitude Scale | -0.05 |
| Psychiatric Diagnosis | -0.05 |
| LEQ Family | -0.05 |
| Truancy | -0.06 |
| Child Experience Rules | -0.06 |
| LEQ Sexuality | -0.07 |
| NEO Conscientiousness | -0.08 |
| NEO Neuroticism | -0.09 |
| ESPAD= European School Survey Project on Alcohol and Other Drugs; LEQ=Life Event Questionnaire; NEO= NEO-Five Factor Personality Inventory; SURPS= Substance Use Risk Profile Scale; TCI=Temperament and Character Inventory | |

| **Table S20. Cortical Surface Area developmental change: Canonical weights (w) of the imaging variables; only non-zero weights are shown** |
| --- |

| **Variables with \|w\|>0.1** |
| --- |

| lh_parstriangularis | 0.63 |
| --- | --- |
| lh_frontalpole | 0.25 |
| lh_pericalcarine | 0.24 |
| rh_superiortemporal | 0.23 |
| rh_pericalcarine | 0.15 |
| lh_middletemporal | 0.14 |
| rh_middletemporal | 0.13 |
| lh_bankssts | -0.27 |
| rh_bankssts | -0.55 |

| **Table S21. Cortical Surface Area developmental change: Canonical loadings (ρ) of the imaging variables** | |
| --- | --- |
| **Variables \|ρ\| > 0.1** | |
| lh_parstriangularis | 0.35 |
| lh_pericalcarine | 0.29 |
| lh_frontalpole | 0.29 |
| rh_superiortemporal | 0.29 |
| rh_pericalcarine | 0.28 |
| lh_middletemporal | 0.27 |
| rh_middletemporal | 0.27 |
| lh_lateraloccipital | 0.25 |
| lh_superiortemporal | 0.25 |
| rh_lateraloccipital | 0.23 |
| rh_frontalpole | 0.23 |
| rh_parstriangularis | 0.22 |
| lh_fusiform | 0.19 |
| lh_superiorfrontal | 0.18 |
| rh_precuneus | 0.18 |
| lh_precuneus | 0.17 |
| rh_lingual | 0.17 |
| rh_paracentral | 0.17 |
| rh_postcentral | 0.17 |
| rh_superiorfrontal | 0.17 |
| lh_medialorbitofrontal | 0.16 |
| lh_parsorbitalis | 0.16 |
| lh_postcentral | 0.16 |
| lh_lingual | 0.15 |
| rh_fusiform | 0.15 |
| rh_inferiortemporal | 0.15 |
| rh_precentral | 0.15 |
| lh_inferiortemporal | 0.14 |
| lh_precentral | 0.14 |
| rh_posteriorcingulate | 0.14 |
| rh_lateralorbitofrontal | 0.12 |
| rh_parsorbitalis | 0.12 |
| lh_lateralorbitofrontal | 0.1 |
| rh_caudalanteriorcingulate | 0.1 |
| rh_cuneus | 0.1 |
| rh_medialorbitofrontal | 0.1 |
| rh_parahippocampal | 0.1 |
| lh_bankssts | -0.29 |
| rh_bankssts | -0.33 |
| **Variables with \|ρ\| < 0.1** | |
| lh_cuneus | 0.09 |
| lh_parahippocampal | 0.09 |
| lh_rostralanteriorcingulate | 0.09 |
| lh_superiorparietal | 0.08 |
| lh_temporalpole | 0.08 |
| rh_temporalpole | 0.08 |
| lh_caudalanteriorcingulate | 0.07 |
| lh_paracentral | 0.07 |
| lh_supramarginal | 0.07 |
| rh_superiorparietal | 0.07 |
| rh_rostralanteriorcingulate | 0.06 |
| lh_parsopercularis | 0.05 |
| lh_posteriorcingulate | 0.05 |
| lh_transversetemporal | 0.05 |
| rh_transversetemporal | 0.04 |
| lh_isthmuscingulate | 0.02 |
| lh_rostralmiddlefrontal | 0.02 |
| rh_entorhinal | 0.02 |
| rh_isthmuscingulate | 0.02 |
| rh_parsopercularis | 0.02 |
| lh_insula | 0.01 |
| rh_inferiorparietal | 0.01 |
| rh_supramarginal | 0.01 |
| rh_insula | 0.01 |
| lh_entorhinal | 0 |
| lh_inferiorparietal | -0.01 |
| rh_caudalmiddlefrontal | -0.01 |
| rh_rostralmiddlefrontal | -0.01 |
| lh_caudalmiddlefrontal | -0.06 |

**4. Sparse Canonical Correlation Analyses: Subcortical Volumes**

**
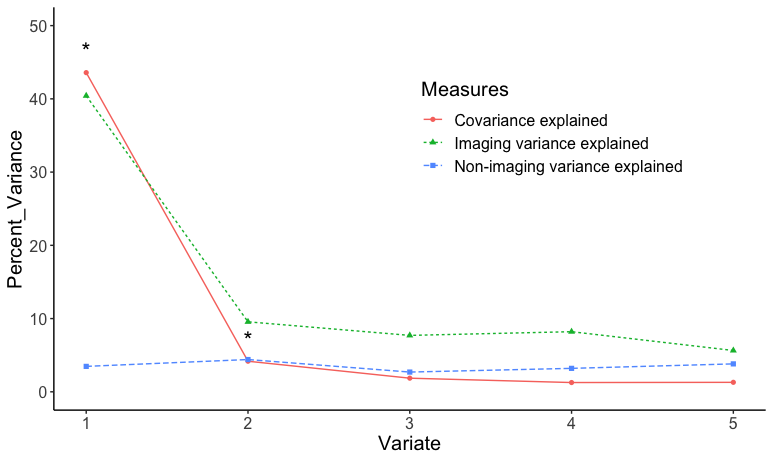
Supplemental Figure S6. Subcortical Volumes at baseline: covariance and variance explained by the canonical variates.** Two canonical mode were statistically significant at an FDR-corrected P value<0.001(*). We reported only the first canonical mode because 1. In testing the parameters of the trained model on the test set only the correlation between the first pair of variates in the test set was at least >80% of that of the training set 2. Only the first mode had a Median RR-Score of >0.80

**Supplemental Figure S7. Subcortical Volumes developmental change: covariance and variance explained by the canonical variates.** One canonical mode was statistically significant at an FDR-corrected P value<0.001 (*). Therefore, we reported only the first canonical mode. In addition 1. In testing the parameters of the trained model on the test set only the correlation between the first pair of variates in the test set was at least >80% of that of the training set 2. Only the first mode had a Median RR-Score of >0.80

**
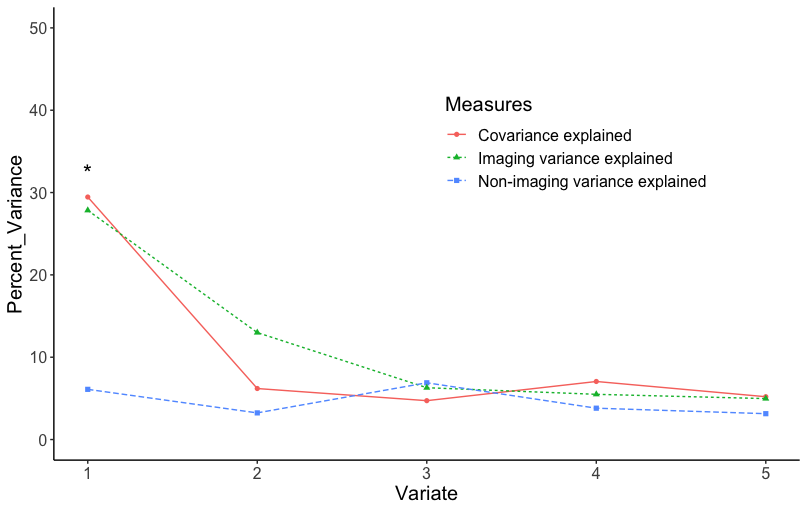
**

| **Table 22.Baseline Subcortical Volume: Canonical weights for non-imaging variables; only non-zero weights are shown** | |
| --- | --- |
| **Variables with \|w\|>0.1** | |
| Sex | 0.88 |
| Height | 0.42 |
| Intelligence g-Factor | 0.17 |
| NEO Neuroticism | -0.11 |
| **Variables with \|w\|<0.1** | |
| Birth Weight | 0.09 |
| SURPS Anxiety Sensitivity | -0.01 |
| Maternal Prenatal Smoking | -0.03 |
| NEO=NEO-Five Factor Personality Inventory | |

| **Table S23. Subcortical Volumes at baseline: Canonical loadings (ρ) for the non-imaging variables** | |
| --- | --- |
| **Variables with \|ρ\|> 0.1** | |
| Sex | 0.62 |
| Height | 0.37 |
| Intelligence g-Factor | 0.23 |
| Birth Weight | 0.19 |
| Weight | 0.14 |
| LEQ Deviance | 0.12 |
| Paternal Education | 0.11 |
| SURPS Sensation Seeking | 0.11 |
| SURPS Anxiety Sensitivity | -0.14 |
| Maternal Prenatal Smoking | -0.15 |
| NEO Neuroticism | -0.2 |
| **Variables \|ρ\| < 0.1** | |
| Perpetrator of Bullying | 0.08 |
| Child Experience Discipline | 0.07 |
| Maternal Education | 0.07 |
| LEQ Autonomy | 0.07 |
| Maternal Prenatal Alcohol Use | 0.06 |
| Pubertal Stage | 0.06 |
| Family Stressors Work/Pressure | 0.05 |
| Average Grade | 0.04 |
| Breastfed | 0.04 |
| ESPAD Frequency of Lifetime Cannabis use | 0.04 |
| Living with Both Parents | 0.03 |
| Family History of Psychiatric Disorders | 0.03 |
| ESPAD Frequency of Cannabis Use in the Preceding Month | 0.03 |
| LEQ Relocation | 0.03 |
| Child Experience Rules | 0.01 |
| Pregnancy Complications | 0.01 |
| Child Experience Affirmation | 0 |
| ESPAD Frequency of Lifetime Alcohol Use | 0 |
| Social Aptitude Scale | -0.01 |
| ESPAD Frequency of Alcohol Use in the Preceding Month | -0.01 |
| LEQ Accident | -0.01 |
| TCI Novelty Seeking | -0.01 |
| Family Stressors Relationship/Addiction | -0.02 |
| ESPAD Frequency of Smoking in the Preceding 30 Days | -0.03 |
| Victim of Bullying | -0.03 |
| NEO Extroversion | -0.03 |
| NEO Openness | -0.03 |
| NEO Conscientiousness | -0.03 |
| LEQ Others | -0.04 |
| NEO Agreeableness | -0.04 |
| SURPS Hopelessness | -0.04 |
| SURPS Impulsivity | -0.04 |
| Age | -0.05 |
| Family Stressors Illness | -0.05 |
| Number of Negative Life Events | -0.05 |
| LEQ Sexuality | -0.05 |
| ESPAD Frequency of ESPAD Frequency of Lifetime Smoking | -0.06 |
| Body Mass Index | -0.06 |
| Family Stressors Socioeconomic/Housing | -0.07 |
| Psychiatric Diagnosis | -0.07 |
| Truancy | -0.07 |
| Maternal Prenatal Medical Illness | -0.08 |
| LEQ Family | -0.08 |
| Paternal Prenatal Smoking | -0.09 |
| LEQ Distress | -0.09 |
| LEQ=Life Event Questionnaire; NEO= NEO-Five Factor Personality Inventory; SURPS= Substance Use Risk Profile Scale; TCI=Temperament and Character Inventory | |

| **Table S24. Subcortical Volumes supplemental at baseline change: Canonical weights (w) of the imaging variables; only non-zero weights are shown** |
| --- |

| **Variables with \|w\|>0.1** |
| --- |

| Estimated Total Intracranial Volume | 0.85 |
| --- | --- |
| Right.Cerebellum.Cortex | 0.36 |
| Left.Cerebellum.Cortex | 0.34 |
| Right.Thalamus.Proper | 0.13 |
| Left.Thalamus.Proper | 0.1 |

| **Variables with \|w\|<0.1** |
| --- |

| Right.VentralDC | 0.05 |
| --- | --- |

| **Table S25. Subcortical Volumes supplemental at baseline: Canonical loadings (ρ) of the imaging variables; all variables had ρ>0.1** | |
| --- | --- |
| Estimated Total Intracranial Volume | 0.61 |
| Left.Cerebellum.Cortex | 0.56 |
| Right.Cerebellum.Cortex | 0.56 |
| Right.Thalamus.Proper | 0.54 |
| Left.Thalamus.Proper | 0.53 |
| Left.VentralDC | 0.48 |
| Right.VentralDC | 0.48 |
| Right.Amygdala | 0.47 |
| Left.Putamen | 0.45 |
| Right.Putamen | 0.45 |
| Left.Amygdala | 0.43 |
| Right.Pallidum | 0.4 |
| Left.Pallidum | 0.39 |
| Left.Hippocampus | 0.39 |
| Right.Hippocampus | 0.37 |
| Right.Caudate | 0.32 |
| Right.Accumbens.area | 0.32 |
| Left.Caudate | 0.31 |
| Left.Accumbens.area | 0.29 |
| Right.Lateral.Ventricle | 0.14 |
| Left.Lateral.Ventricle | 0.13 |

| **Table S26. Subcortical Volumes developmental change: Canonical weights (w) of the non-imaging variables; only non-zero weights are shown** | |
| --- | --- |
| **Variables with \|w\|>0.1** | |
| Height | 0.63 |
| Sex | 0.62 |
| Weight | 0.43 |
| **Variables with \|w\|<0.1** | |
| Age | 0.08 |
| Body Mass Index | 0.05 |
| SURPS Sensation Seeking | 0.01 |
| Number of Negative Life Events | -0.01 |
| NEO Extroversion | -0.02 |
| Child Experience Rules | -0.03 |
| Child Experience Affirmation | -0.04 |
| LEQ Accident | -0.07 |
| NEO Conscientiousness | -0.08 |
| SURPS Anxiety Sensitivity | -0.08 |
| LEQ Sexuality | -0.09 |
| LEQ=Life Event Questionnaire; NEO=NEO-Five Factor Personality Inventory | |

| **Table S27** **Subcortical Volumes developmental change: Canonical loadings (ρ) for the non-imaging variables** | |
| --- | --- |
| **Variables with \|ρ\| > 0.1** | |
| Sex | 0.48 |
| Height | 0.48 |
| Weight | 0.35 |
| Age | 0.12 |
| Body Mass Index | 0.1 |
| Child Experience Affirmation | -0.1 |
| LEQ Accident | -0.11 |
| NEO Conscientiousness | -0.12 |
| SURPS Anxiety Sensitivity | -0.12 |
| LEQ Sexuality | -0.13 |
| **Variables with \|ρ\| < 0.1** | |
| ESPAD Frequency of Alcohol Use in the Preceding Month | 0.07 |
| ESPAD Frequency of Lifetime Cannabis use | 0.07 |
| SURPS Sensation Seeking | 0.07 |
| Family Stressors Work/Pressure | 0.06 |
| ESPAD Frequency of Lifetime Alcohol Use | 0.05 |
| LEQ Deviance | 0.05 |
| LEQ Others | 0.05 |
| Perpetrator of Bullying | 0.04 |
| LEQ Autonomy | 0.04 |
| SURPS Hopelessness | 0.04 |
| ESPAD Frequency of Cannabis Use in the Preceding Month | 0.03 |
| LEQ Relocation | 0.03 |
| ESPAD Frequency of Smoking in the Preceding Month | 0.01 |
| Average Grade | 0.01 |
| LEQ Family | 0.01 |
| SURPS Impulsivity | 0 |
| TCI Novelty Seeking | 0 |
| Living with Both Parents | -0.01 |
| Family Stressors Socioeconomic/Housing | -0.01 |
| ESPAD Frequency of Lifetime Smoking | -0.01 |
| NEO Openness | -0.01 |
| Family Stressors Relationship/Addiction | -0.02 |
| Child Experience Discipline | -0.02 |
| Victim of Bullying | -0.02 |
| LEQ Distress | -0.02 |
| Family Stressors Illness | -0.04 |
| Social Aptitude Scale | -0.04 |
| NEO Agreeableness | -0.04 |
| Psychiatric Diagnosis | -0.05 |
| Truancy | -0.05 |
| NEO Neuroticism | -0.06 |
| Number of Negative Life Events | -0.07 |
| NEO Extroversion | -0.08 |
| Child Experience Rules | -0.09 |
| ESPAD= European School Survey Project on Alcohol and Other Drugs; LEQ=Life Event Questionnaire; NEO= NEO-Five Factor Personality Inventory; SURPS= Substance Use Risk Profile Scale; TCI=Temperament and Character Inventory | |

| **Table S28. Subcortical Volumes developmental change: Canonical weights (w) of the imaging variables; only non-zero weights are shown** | |
| --- | --- |
| **Variables with \|w\|>0.1** | |
| Left.VentralDC | 0.45 |
| Right.VentralDC | 0.44 |
| Right.Caudate | 0.39 |
| Right.Hippocampus | 0.31 |
| Left.Pallidum | 0.3 |
| Left.Hippocampus | 0.3 |
| Left.Caudate | 0.25 |
| Right.Pallidum | 0.2 |
| Right.Amygdala | 0.17 |
| Right.Putamen | 0.15 |
| Left.Amygdala | 0.11 |
| Left.Accumbens.area | 0.1 |
| **Variables with \|w\|<0.1** | |
| Left.Putamen | 0.07 |
| Right.Cerebellum.Cortex | 0.06 |

| **Table S29. Subcortical Volumes developmental change: Canonical loadings (ρ) of the imaging variables** | |
| --- | --- |
| **Variables with \|ρ\| > 0.1** | |
| Left.VentralDC | 0.4 |
| Right.VentralDC | 0.4 |
| Right.Caudate | 0.38 |
| Right.Hippocampus | 0.34 |
| Left.Pallidum | 0.33 |
| Left.Hippocampus | 0.33 |
| Left.Caudate | 0.31 |
| Right.Pallidum | 0.28 |
| Right.Amygdala | 0.27 |
| Right.Putamen | 0.26 |
| Left.Amygdala | 0.24 |
| Left.Accumbens.area | 0.24 |
| Left.Putamen | 0.22 |
| Right.Cerebellum.Cortex | 0.22 |
| Left.Thalamus.Proper | 0.19 |
| Right.Thalamus.Proper | 0.18 |
| Right.Accumbens.area | 0.18 |
| Left.Cerebellum.Cortex | 0.17 |
| Right.Lateral.Ventricle | 0.11 |
| Left.Lateral.Ventricle | 0.1 |

1. **Reliability and Reproducibility**

The correlations between image quality and the cortical thickness supplemental coefficients was not significant (Spearman’s Rho=-0.02, p= 0.72).

**Supplemental Figure S8. Reliability of the results as a function of sample size**

**A. Baseline**


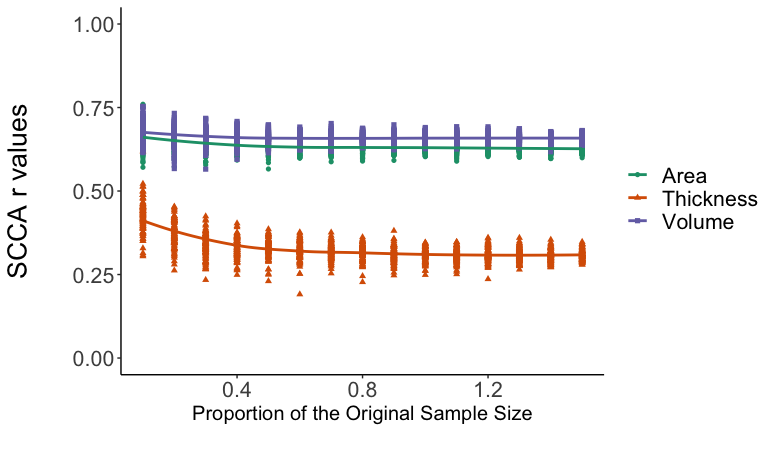


**B. Developmental Change**

**
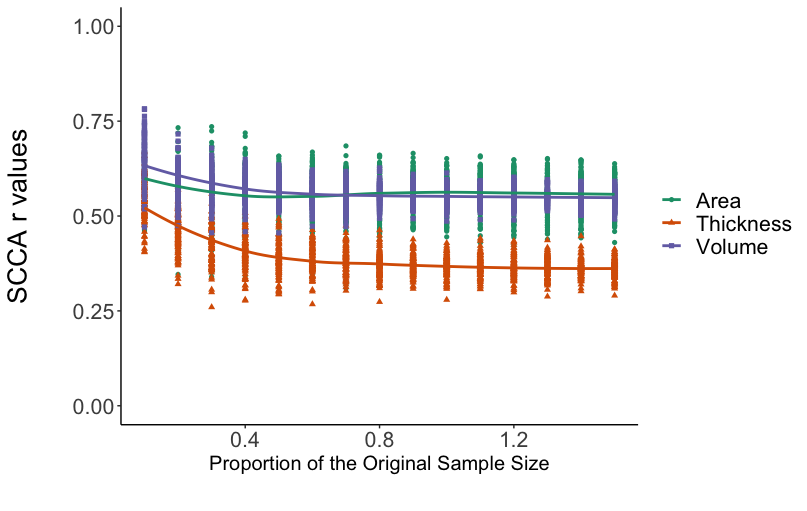
**

**Supplemental Figure S8. Redundancy Reliability Scores of the first mode for each supplemental**

**A.Baseline**


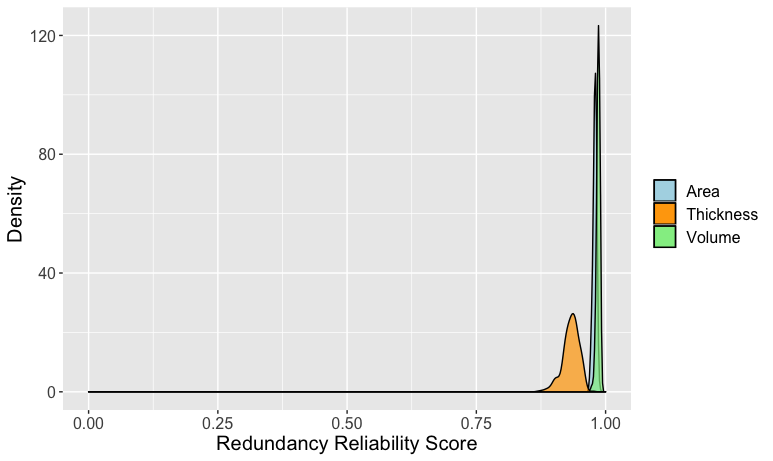


**B. Developmental Change**


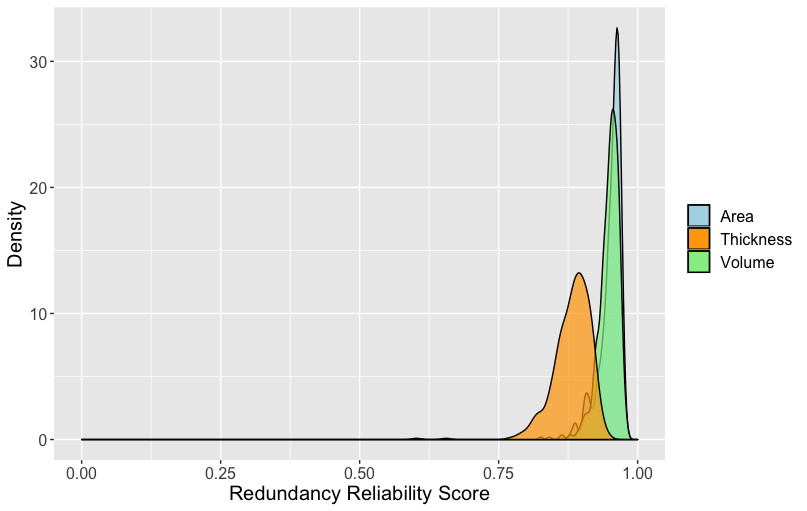


| **Table S30. Mean and standard deviations of supplemental Correlation coefficients for the first five modes in 500 test/train sets** | | | | | | |
| --- | --- | --- | --- | --- | --- | --- |
| **Baseline** | | | | | | |
| **Modes** | **Thickness** | | **Surface Area** | | **Volume** | |
|  | **Train** | **Test** | **Train** | **Test** | **Train** | **Test** |
| **1** | 0.28(0.01) | 0.26(0.03) | 0.62(0.01) | 0.62(0.02) | 0.65(0.01) | 0.65(0.02) |
| **2** | 0.19(0.03) | 0.11(0.05) | 0.12(0.04) | 0.02(0.06) | 0.2(0.06) | 0.17(0.08) |
| **3** | 0.15(0.03) | 0.07(0.05) | 0.14(0.04) | 0.05(0.07) | 0.1(0.07) | 0.05(0.09) |
| **4** | 0.15(0.03) | 0.06(0.05) | 0.14(0.04) | 0.04(0.06) | 0.09(0.05) | 0.03(0.07) |
| **5** | 0.14(0.03) | 0.05(0.05) | 0.13(0.05) | 0.03(0.06) | 0.09(0.06) | 0.05(0.08) |
| **Developmental Change** | | | | | | |
| **Modes** | **Thickness** | | **Surface Area** | | **Volume** | |
|  | **Train** | **Test** | **Train** | **Test** | **Train** | **Test** |
| **1** | 0.33(0.01) | 0.29(0.03) | 0.52(0.07) | 0.48(0.09) | 0.53(0.01) | 0.51(0.04) |
| **2** | 0.23(0.11) | 0.14(0.14) | 0.17(0.06) | 0.05(0.08) | 0.15(0.03) | 0.04(0.05) |
| **3** | 0.19(0.11) | 0.07(0.15) | 0.16(0.06) | 0.05(0.09) | 0.16(0.03) | 0.05(0.05) |
| **4** | 0.14(0.06) | 0(0.09) | 0.16(0.06) | 0.05(0.09) | 0.15(0.04) | 0.04(0.06) |
| **5** | 0.14(0.04) | -0.01(0.06) | 0.15(0.05) | 0.03(0.07) | 0.15(0.04) | 0.03(0.06) |

| **Table S31. False discovery rate adjusted P-values of the first five SCCA modes after regressing out sex and age** | | | |
| --- | --- | --- | --- |
| **Baseline** | | | |
| **Modes** | **Thickness** | **Surface Area** | **Volume** |
| **1** | 0.107 | 0 | 0 |
| **2** | 0.100 | 0.100 | 0.923 |
| **3** | 0.217 | 0.632 | 0.923 |
| **4** | 0.161 | 0.430 | 0.530 |
| **5** | 0.125 | 0.494 | 0.923 |
| **Developmental Change** | | | |
| **Modes** | **Thickness** | **Surface Area** | **Volume** |
| **1** | 0.045 | 0 | 0 |
| **2** | 0.595 | 0.010 | 0.251 |
| **3** | 0.836 | 0.317 | 0.359 |
| **4** | 0.898 | 0.589 | 0.0783 |
| **5** | 0.836 | 0.589 | 0.005 |

**Top variable-variate correlations for the first significant modes**

**Below we show the top non-imaging and imaging variable-to-variate correlations. Positive correlations are indicated by a plus sign (+) and negative correlations by a negative sign (-)**

**Cortical Thickness-Baseline: The results were not significant**

**Cortical Surface Area-Baseline**

Top non-imaging correlations: Intelligence (+), height (+), birthweight (+), parental education (+), parental perinatal smoking (-)

Top imaging variable-non-imaging variate correlations: total area (+), bilateral lateral orbitofrontal (+), bilateral insular (+), bilateral middle temporal (+), bilateral medial orbitofrontal (+)

**Subcortical Volumes-Baseline**

Top non-imaging correlations: Height (+), Intelligence (+), Birthweight (+), weight (+), pubertal stage (+)

Top imaging correlations: total intracranial volume (+), bilateral ventral DC (+), bilateral thalami (+), left hippocampus (+), right amygdala (+)

**Cortical Thickness-Developmental Change**

Top non-imaging variable- imaging variate correlations: Monthly drinking (+); Top imaging variable-non-imaging variate correlations: bilateral superior frontal (-), bilateral caudal middle frontal (-), bilateral rostral middle frontal (-), left precentral (-), left supramarginal (-)

**Cortical Surface Area-Developmental Change**

Top non-imaging variable- imaging variate correlations: Height (+), agreeableness (+), extraversion (-); Top imaging variable-non-imaging variate correlations: Bilateral middle temporal (+), bilateral lateral occipital (+), left pars triangularis (-), left pericalcarine (+), right superior temporal (+)

**Subcortical Volume-Developmental Change**

Top non-imaging variable- imaging variate correlations: Height (+), weight (+); Top imaging variable-non-imaging variate correlations: bilateral ventral DC (+), bilateral pallidum (+), bilateral caudate (+), bilateral amygdala (+)

| **Table S32. False discovery rate adjusted P-values of the first five SCCA modes of developmental change data after including baseline only variables** | | | |
| --- | --- | --- | --- |
| **Modes** | **Thickness** | **Surface Area** | **Volume** |
| **1** | 0 | 0 | 0 |
| **2** | 0 | 0.193 | 0.601 |
| **3** | 0.760 | 0 | 0.601 |
| **4** | 0.296 | 0.765 | 0.027 |
| **5** | 0.013 | 0.892 | 0.601 |

**Top variable-variate correlations for the first significant modes**

**Below we show the top non-imaging and imaging variable-to-variate correlations. Positive correlations are indicated by a plus sign (+) and negative correlations by a negative sign (-)**

**Cortical Thickness**

Top five significant non-imaging variable- imaging variate correlations: Age (-), Alcohol Use in the preceding 30 days (-), height (-), pubertal stage (+), Cannabis Use in the preceding 30 days (-)

Top five significant imaging variable-non-imaging variate correlations: bilateral (+)

Posterior cingulate (+), superior frontal (+), pars opercularis (+), banks of the superior temporal sulcus (+), supramarginal (+)

**Cortical Surface Area**

Top five significant non-imaging variable- imaging variate correlations: height (+), sex (+), weight (+), age (+), pubertal stage (-)

Additional significant baseline variables: maternal education (+), breastfeeding (-)

Top five significant imaging variable-non-imaging variate correlations: Bilateral pars triangularis (+), bilateral frontal pole (+), bilateral pericalcarine (+), bilateral superior temporal (+), bilateral middle temporal (+)

**Subcortical Volume**

Top five significant non-imaging variable- imaging variate correlations: Sex (+), height (+), weight (+), maternal education (+), conscientiousness (-)

Additional significant baseline variables: breastfeeding (-)

Top five significant imaging variable-non-imaging variate correlations: Bilateral caudate (+), ventral DC (+), hippocampus (+), putamen (+), pallidum (+)

**References**

1. Furnham A, Guenole N, Levine SZ, Chamorro-Premuzic T. The NEO Personality Inventory-Revised: factor structure and gender invariance from exploratory structural equation modeling analyses in a high-stakes setting. *Assessment* 2013; **20**(1)**:** 14-23.

2. Schumann G, Loth E, Banaschewski T, Barbot A, Barker G, Buchel C *et al.* The IMAGEN study: reinforcement-related behaviour in normal brain function and psychopathology. *Mol Psychiatry* 2010; **15**(12)**:** 1128-1139.

3. Klapwijk ET, van de Kamp F, van der Meulen M, Peters S, Wierenga LM. Qoala-T: A supervised-learning tool for quality control of FreeSurfer segmented MRI data. *Neuroimage* 2019; **189:** 116-129.

4. Reuter M, Schmansky NJ, Rosas HD, Fischl B. Within-subject template estimation for unbiased longitudinal image analysis. *Neuroimage* 2012; **61**(4)**:** 1402-1418.

5. Wang H-T, Smallwood J, Mourao-Miranda J, Xia CH, Satterthwaite TD, Bassett DS *et al.* Finding the needle in high-dimensional haystack: A tutorial on canonical correlation analysis. 2018.

6. On the equivalence between canonical correlation analysis and orthonormalized partial least squares. *Proceedings of the Twenty-First International Joint Conference on Artificial Intelligence*2009.

7. Tibshirani R. The lasso method for variable selection in the Cox model. *Stat Med* 1997; **16**(4)**:** 385-395.

8. Witten DM, Tibshirani R, Hastie T. A penalized matrix decomposition, with applications to sparse principal components and canonical correlation analysis. *Biostatistics* 2009; **10**(3)**:** 515-534.

9. Cohen J. *Statistical power analysis for the behavioral sciences*. Routledge2013.

10. Moser DA, Doucet GE, Lee WH, Rasgon A, Krinsky H, Leibu E *et al.* Multivariate Associations Among Behavioral, Clinical, and Multimodal Imaging Phenotypes in Patients With Psychosis. *JAMA Psychiatry* 2018; **75**(4)**:** 386-395.

**List of publications by the IMAGEN Consortium**

1 Albaugh, M. D. et al. White matter microstructure is associated with hyperactive/inattentive symptomatology and polygenic risk for attention-deficit/hyperactivity disorder in a population-based sample of adolescents. Neuropsychopharmacology : official publication of the American College of Neuropsychopharmacology 44, 1597-1603, doi:10.1038/s41386-019-0383-y (2019).

2 Albaugh, M. D. et al. Amygdalar reactivity is associated with prefrontal cortical thickness in a large population-based sample of adolescents. PloS one 14, e0216152, doi:10.1371/journal.pone.0216152 (2019).

3 Baker, T. E. et al. Modulation of orbitofrontal-striatal reward activity by dopaminergic functional polymorphisms contributes to a predisposition to alcohol misuse in early adolescence. Psychological medicine 49, 801-810, doi:10.1017/s0033291718001459 (2019).

4 Barker, E. D. et al. Do ADHD-impulsivity and BMI have shared polygenic and neural correlates? Molecular psychiatry, doi:10.1038/s41380-019-0444-y (2019).

5 Bartholdy, S. et al. Neural Correlates of Failed Inhibitory Control as an Early Marker of Disordered Eating in Adolescents. Biological psychiatry 85, 956-965, doi:10.1016/j.biopsych.2019.01.027 (2019).

6 Bayard, F. et al. Distinct brain structure and behavior related to ADHD and conduct disorder traits. Molecular psychiatry, doi:10.1038/s41380-018-0202-6 (2018).

7 Bossier, H. et al. The empirical replicability of task-based fMRI as a function of sample size. NeuroImage 212, 116601, doi:10.1016/j.neuroimage.2020.116601 (2020).

8 Bourque, J. et al. Functional Neuroimaging Predictors of Self-Reported Psychotic Symptoms in Adolescents. The American journal of psychiatry 174, 566-575, doi:10.1176/appi.ajp.2017.16080897 (2017).

9 Brislin, S. J. et al. Extending the Construct Network of Trait Disinhibition to the Neuroimaging Domain: Validation of a Bridging Scale for Use in the European IMAGEN Project. Assessment 26, 567-581, doi:10.1177/1073191118759748 (2019).

10 Buchel, C. et al. Blunted ventral striatal responses to anticipated rewards foreshadow problematic drug use in novelty-seeking adolescents. Nature communications 8, 14140, doi:10.1038/ncomms14140 (2017).

11 Burt, K. B. et al. Structural brain correlates of adolescent resilience. Journal of child psychology and psychiatry, and allied disciplines 57, 1287-1296, doi:10.1111/jcpp.12552 (2016).

12 Cao, Z. et al. Mapping adolescent reward anticipation, receipt, and prediction error during the monetary incentive delay task. Human brain mapping 40, 262-283, doi:10.1002/hbm.24370 (2019).

13 Castellanos-Ryan, N. et al. The structure of psychopathology in adolescence and its common personality and cognitive correlates. Journal of abnormal psychology 125, 1039-1052, doi:10.1037/abn0000193 (2016).

14 Castellanos-Ryan, N. et al. Neural and cognitive correlates of the common and specific variance across externalizing problems in young adolescence. The American journal of psychiatry 171, 1310-1319, doi:10.1176/appi.ajp.2014.13111499 (2014).

15 Chaarani, B. et al. Low Smoking Exposure, the Adolescent Brain, and the Modulating Role of CHRNA5 Polymorphisms. Biological psychiatry. Cognitive neuroscience and neuroimaging 4, 672-679, doi:10.1016/j.bpsc.2019.02.006 (2019).

16 Cheng, W. et al. Decreased brain connectivity in smoking contrasts with increased connectivity in drinking. eLife 8, doi:10.7554/eLife.40765 (2019).

17 Cury, C. et al. Statistical Shape Analysis of Large Datasets Based on Diffeomorphic Iterative Centroids. Frontiers in neuroscience 12, 803, doi:10.3389/fnins.2018.00803 (2018).

18 Cury, C. et al. Genome wide association study of incomplete hippocampal inversion in adolescents. PloS one 15, e0227355, doi:10.1371/journal.pone.0227355 (2020).

19 Cury, C. et al. Incomplete Hippocampal Inversion: A Comprehensive MRI Study of Over 2000 Subjects. Frontiers in neuroanatomy 9, 160, doi:10.3389/fnana.2015.00160 (2015).

20 Davids, M. et al. Fully-automated quality assurance in multi-center studies using MRI phantom measurements. Magnetic resonance imaging 32, 771-780, doi:10.1016/j.mri.2014.01.017 (2014).

21 Dell'Acqua, F. et al. Tract Based Spatial Statistic Reveals No Differences in White Matter Microstructural Organization between Carriers and Non-Carriers of the APOE varepsilon4 and varepsilon2 Alleles in Young Healthy Adolescents. Journal of Alzheimer's disease : JAD 47, 977-984, doi:10.3233/jad-140519 (2015).

22 Deng, W. et al. Separate neural systems for behavioral change and for emotional responses to failure during behavioral inhibition. Human brain mapping 38, 3527-3537, doi:10.1002/hbm.23607 (2017).

23 Desrivieres, S. et al. Single nucleotide polymorphism in the neuroplastin locus associates with cortical thickness and intellectual ability in adolescents. Molecular psychiatry 20, 263-274, doi:10.1038/mp.2013.197 (2015).

24 Dickie, E. W. et al. Global genetic variations predict brain response to faces. PLoS genetics 10, e1004523, doi:10.1371/journal.pgen.1004523 (2014).

25 Duka, T. et al. GABRB1 Single Nucleotide Polymorphism Associated with Altered Brain Responses (but not Performance) during Measures of Impulsivity and Reward Sensitivity in Human Adolescents. Frontiers in behavioral neuroscience 11, 24, doi:10.3389/fnbeh.2017.00024 (2017).

26 Ernst, M. et al. Pubertal maturation and sex effects on the default-mode network connectivity implicated in mood dysregulation. Translational psychiatry 9, 103, doi:10.1038/s41398-019-0433-6 (2019).

27 Ewald, A. et al. The role of the cannabinoid receptor in adolescents' processing of facial expressions. The European journal of neuroscience 43, 98-105, doi:10.1111/ejn.13118 (2016).

28 French, L. et al. Early Cannabis Use, Polygenic Risk Score for Schizophrenia and Brain Maturation in Adolescence. JAMA psychiatry 72, 1002-1011, doi:10.1001/jamapsychiatry.2015.1131 (2015).

29 Frere, P. B. et al. Sex effects on structural maturation of the limbic system and outcomes on emotional regulation during adolescence. NeuroImage 210, 116441, doi:10.1016/j.neuroimage.2019.116441 (2020).

30 Fritsch, V. et al. Robust regression for large-scale neuroimaging studies. NeuroImage 111, 431-441, doi:10.1016/j.neuroimage.2015.02.048 (2015).

31 Galinowski, A. et al. Heavy drinking in adolescents is associated with change in brainstem microstructure and reward sensitivity. Addiction biology, e12781, doi:10.1111/adb.12781 (2019).

32 Galinowski, A. et al. Resilience and corpus callosum microstructure in adolescence. Psychological medicine 45, 2285-2294, doi:10.1017/s0033291715000239 (2015).

33 Gollier-Briant, F. et al. Neural correlates of three types of negative life events during angry face processing in adolescents. Social cognitive and affective neuroscience 11, 1961-1969, doi:10.1093/scan/nsw100 (2016).

34 Gonzalez, D. A. et al. The Arf6 activator Efa6/PSD3 confers regional specificity and modulates ethanol consumption in Drosophila and humans. Molecular psychiatry 23, 621-628, doi:10.1038/mp.2017.112 (2018).

35 Grasby, K. L. et al. The genetic architecture of the human cerebral cortex. Science (New York, N.Y.) 367, doi:10.1126/science.aay6690 (2020).

36 Grigis, A. et al. Neuroimaging, Genetics, and Clinical Data Sharing in Python Using the CubicWeb Framework. Frontiers in neuroinformatics 11, 18, doi:10.3389/fninf.2017.00018 (2017).

37 Hass, J. et al. A Genome-Wide Association Study Suggests Novel Loci Associated with a Schizophrenia-Related Brain-Based Phenotype. PloS one 8, e64872, doi:10.1371/journal.pone.0064872 (2013).

38 Heinrich, A. et al. The risk variant in ODZ4 for bipolar disorder impacts on amygdala activation during reward processing. Bipolar disorders 15, 440-445, doi:10.1111/bdi.12068 (2013).

39 Heinrich, A. et al. Prediction of alcohol drinking in adolescents: Personality-traits, behavior, brain responses, and genetic variations in the context of reward sensitivity. Biological psychology 118, 79-87, doi:10.1016/j.biopsycho.2016.05.002 (2016).

40 Heinrich, A. et al. From gene to brain to behavior: schizophrenia-associated variation in AMBRA1 alters impulsivity-related traits. The European journal of neuroscience 38, 2941-2945, doi:10.1111/ejn.12201 (2013).

41 Heinrich, A., Schumann, G., Flor, H. & Nees, F. Identification of Key Items Regarding Personality, Environment, and Life Events to Assess Risk and Resilience Factors for Harmful Alcohol Drinking in Adolescents. Alcohol and alcoholism (Oxford, Oxfordshire) 51, 710-715, doi:10.1093/alcalc/agw012 (2016).

42 Hibar, D. P. et al. Common genetic variants influence human subcortical brain structures. Nature 520, 224-229, doi:10.1038/nature14101 (2015).

43 Huguet, G. et al. Measuring and Estimating the Effect Sizes of Copy Number Variants on General Intelligence in Community-Based Samples. JAMA psychiatry 75, 447-457, doi:10.1001/jamapsychiatry.2018.0039 (2018).

44 Ing, A. et al. Identification of neurobehavioural symptom groups based on shared brain mechanisms. Nature human behaviour 3, 1306-1318, doi:10.1038/s41562-019-0738-8 (2019).

45 Jia, T. et al. Neural basis of reward anticipation and its genetic determinants. Proceedings of the National Academy of Sciences of the United States of America 113, 3879-3884, doi:10.1073/pnas.1503252113 (2016).

46 Jurk, S. et al. Personality and substance use: psychometric evaluation and validation of the Substance Use Risk Profile Scale (SURPS) in English, Irish, French, and German adolescents. Alcoholism, clinical and experimental research 39, 2234-2248, doi:10.1111/acer.12886 (2015).

47 Kaminski, J. A. et al. Epigenetic variance in dopamine D2 receptor: a marker of IQ malleability? Translational psychiatry 8, 169, doi:10.1038/s41398-018-0222-7 (2018).

48 Khan, W. et al. A Multi-Cohort Study of ApoE varepsilon4 and Amyloid-beta Effects on the Hippocampus in Alzheimer's Disease. Journal of Alzheimer's disease : JAD 56, 1159-1174, doi:10.3233/jad-161097 (2017).

49 Khan, W. et al. No differences in hippocampal volume between carriers and non-carriers of the ApoE epsilon4 and epsilon2 alleles in young healthy adolescents. Journal of Alzheimer's disease : JAD 40, 37-43, doi:10.3233/jad-131841 (2014).

50 Kuhn, S. et al. Hierarchical associations of alcohol use disorder symptoms in late adolescence with markers during early adolescence. Addictive behaviors 100, 106130, doi:10.1016/j.addbeh.2019.106130 (2020).

51 Kuhn, S. et al. Positive association of video game playing with left frontal cortical thickness in adolescents. PloS one 9, e91506, doi:10.1371/journal.pone.0091506 (2014).

52 Kuhn, S. et al. Predicting development of adolescent drinking behaviour from whole brain structure at 14 years of age. eLife 8, doi:10.7554/eLife.44056 (2019).

53 Kuhn, S. et al. Predicting change trajectories of neuroticism from baseline brain structure using whole brain analyses and latent growth curve models in adolescents. Scientific reports 10, 1207, doi:10.1038/s41598-020-58128-x (2020).

54 Kuhn, S. et al. Manual dexterity correlating with right lobule VI volume in right-handed 14-year-olds. NeuroImage 59, 1615-1621, doi:10.1016/j.neuroimage.2011.08.100 (2012).

55 Kuhn, S. et al. From mother to child: orbitofrontal cortex gyrification and changes of drinking behaviour during adolescence. Addiction biology 21, 700-708, doi:10.1111/adb.12240 (2016).

56 Lancaster, T. M. et al. Polygenic Risk of Psychosis and Ventral Striatal Activation During Reward Processing in Healthy Adolescents. JAMA psychiatry 73, 852-861, doi:10.1001/jamapsychiatry.2016.1135 (2016).

57 Lett, T. A. et al. Cortical Surfaces Mediate the Relationship Between Polygenic Scores for Intelligence and General Intelligence. Cerebral cortex (New York, N.Y. : 1991), doi:10.1093/cercor/bhz270 (2019).

58 Loth, E. et al. Oxytocin receptor genotype modulates ventral striatal activity to social cues and response to stressful life events. Biological psychiatry 76, 367-376, doi:10.1016/j.biopsych.2013.07.043 (2014).

59 Luo, Q. et al. Association of a Schizophrenia-Risk Nonsynonymous Variant With Putamen Volume in Adolescents: A Voxelwise and Genome-Wide Association Study. JAMA psychiatry 76, 435-445, doi:10.1001/jamapsychiatry.2018.4126 (2019).

60 Macare, C. et al. A neurobiological pathway to smoking in adolescence: TTC12-ANKK1-DRD2 variants and reward response. European neuropsychopharmacology : the journal of the European College of Neuropsychopharmacology 28, 1103-1114, doi:10.1016/j.euroneuro.2018.07.101 (2018).

61 Mackey, S. et al. Brain Regions Related to Impulsivity Mediate the Effects of Early Adversity on Antisocial Behavior. Biological psychiatry 82, 275-282, doi:10.1016/j.biopsych.2015.12.027 (2017).

62 Mareckova, K. et al. Hormonal contraceptives, menstrual cycle and brain response to faces. Social cognitive and affective neuroscience 9, 191-200, doi:10.1093/scan/nss128 (2014).

63 Meng, W. et al. Genotype-dependent epigenetic regulation of DLGAP2 in alcohol use and dependence. Molecular psychiatry, doi:10.1038/s41380-019-0588-9 (2019).

64 Mielenz, D. et al. EFhd2/Swiprosin-1 is a common genetic determinator for sensation-seeking/low anxiety and alcohol addiction. Molecular psychiatry 23, 1303-1319, doi:10.1038/mp.2017.63 (2018).

65 Mikita, N. et al. Disentangling the autism-anxiety overlap: fMRI of reward processing in a community-based longitudinal study. Translational psychiatry 6, e845, doi:10.1038/tp.2016.107 (2016).

66 Miller, M. L. et al. Ventral striatal regulation of CREM mediates impulsive action and drug addiction vulnerability. Molecular psychiatry 23, 1328-1335, doi:10.1038/mp.2017.80 (2018).

67 Montigny, C. et al. A phenotypic structure and neural correlates of compulsive behaviors in adolescents. PloS one 8, e80151, doi:10.1371/journal.pone.0080151 (2013).

68 Muller, K. U. et al. No differences in ventral striatum responsivity between adolescents with a positive family history of alcoholism and controls. Addiction biology 20, 534-545, doi:10.1111/adb.12136 (2015).

69 Muller, K. U. et al. Altered reward processing in adolescents with prenatal exposure to maternal cigarette smoking. JAMA psychiatry 70, 847-856, doi:10.1001/jamapsychiatry.2013.44 (2013).

70 Nees, F. et al. Brain substrates of reward processing and the mu-opioid receptor: a pathway into pain? Pain 158, 212-219, doi:10.1097/j.pain.0000000000000720 (2017).

71 Nees, F. et al. Determinants of early alcohol use in healthy adolescents: the differential contribution of neuroimaging and psychological factors. Neuropsychopharmacology : official publication of the American College of Neuropsychopharmacology 37, 986-995, doi:10.1038/npp.2011.282 (2012).

72 Nees, F. et al. A target sample of adolescents and reward processing: same neural and behavioral correlates engaged in common paradigms? Experimental brain research 223, 429-439, doi:10.1007/s00221-012-3272-8 (2012).

73 Nees, F. et al. BDNF Val66Met and reward-related brain function in adolescents: role for early alcohol consumption. Alcohol (Fayetteville, N.Y.) 49, 103-110, doi:10.1016/j.alcohol.2014.12.004 (2015).

74 Nees, F. et al. Genetic risk for nicotine dependence in the cholinergic system and activation of the brain reward system in healthy adolescents. Neuropsychopharmacology : official publication of the American College of Neuropsychopharmacology 38, 2081-2089, doi:10.1038/npp.2013.131 (2013).

75 Nemmi, F. et al. Interaction between striatal volume and DAT1 polymorphism predicts working memory development during adolescence. Developmental cognitive neuroscience 30, 191-199, doi:10.1016/j.dcn.2018.03.006 (2018).

76 Nymberg, C. et al. DRD2/ANKK1 polymorphism modulates the effect of ventral striatal activation on working memory performance. Neuropsychopharmacology : official publication of the American College of Neuropsychopharmacology 39, 2357-2365, doi:10.1038/npp.2014.83 (2014).

77 Nymberg, C. et al. Neural mechanisms of attention-deficit/hyperactivity disorder symptoms are stratified by MAOA genotype. Biological psychiatry 74, 607-614, doi:10.1016/j.biopsych.2013.03.027 (2013).

78 Nymberg, C., Jia, T., Ruggeri, B. & Schumann, G. Analytical strategies for large imaging genetic datasets: experiences from the IMAGEN study. Annals of the New York Academy of Sciences 1282, 92-106, doi:10.1111/nyas.12088 (2013).

79 Ojelade, S. A. et al. Rsu1 regulates ethanol consumption in Drosophila and humans. Proceedings of the National Academy of Sciences of the United States of America 112, E4085-4093, doi:10.1073/pnas.1417222112 (2015).

80 O'Leary-Barrett, M. et al. Personality, Attentional Biases towards Emotional Faces and Symptoms of Mental Disorders in an Adolescent Sample. PloS one 10, e0128271, doi:10.1371/journal.pone.0128271 (2015).

81 Ortuno-Sierra, J. et al. New evidence of factor structure and measurement invariance of the SDQ across five European nations. European child & adolescent psychiatry 24, 1523-1534, doi:10.1007/s00787-015-0729-x (2015).

82 Paillere Martinot, M. L. et al. White-matter microstructure and gray-matter volumes in adolescents with subthreshold bipolar symptoms. Molecular psychiatry 19, 462-470, doi:10.1038/mp.2013.44 (2014).

83 Papanastasiou, E. et al. Examination of the Neural Basis of Psychoticlike Experiences in Adolescence During Reward Processing. JAMA psychiatry 75, 1043-1051, doi:10.1001/jamapsychiatry.2018.1973 (2018).

84 Pena-Oliver, Y. et al. Mouse and Human Genetic Analyses Associate Kalirin with Ventral Striatal Activation during Impulsivity and with Alcohol Misuse. Frontiers in genetics 7, 52, doi:10.3389/fgene.2016.00052 (2016).

85 Peters, J. et al. Lower ventral striatal activation during reward anticipation in adolescent smokers. The American journal of psychiatry 168, 540-549, doi:10.1176/appi.ajp.2010.10071024 (2011).

86 Quinlan, E. B. et al. Identifying biological markers for improved precision medicine in psychiatry. Molecular psychiatry 25, 243-253, doi:10.1038/s41380-019-0555-5 (2020).

87 Quinlan, E. B. et al. Peer victimization and its impact on adolescent brain development and psychopathology. Molecular psychiatry, doi:10.1038/s41380-018-0297-9 (2018).

88 Quinlan, E. B. et al. Psychosocial Stress and Brain Function in Adolescent Psychopathology. The American journal of psychiatry 174, 785-794, doi:10.1176/appi.ajp.2017.16040464 (2017).

89 Richiardi, J. et al. BRAIN NETWORKS. Correlated gene expression supports synchronous activity in brain networks. Science (New York, N.Y.) 348, 1241-1244, doi:10.1126/science.1255905 (2015).

90 Robert, G. H. et al. Association of Gray Matter and Personality Development With Increased Drunkenness Frequency During Adolescence. JAMA psychiatry, doi:10.1001/jamapsychiatry.2019.4063 (2019).

91 Ruan, H. et al. Adolescent binge drinking disrupts normal trajectories of brain functional organization and personality maturation. NeuroImage. Clinical 22, 101804, doi:10.1016/j.nicl.2019.101804 (2019).

92 Ruggeri, B. et al. Methylation of OPRL1 mediates the effect of psychosocial stress on binge drinking in adolescents. Journal of child psychology and psychiatry, and allied disciplines 59, 650-658, doi:10.1111/jcpp.12843 (2018).

93 Ruggeri, B. et al. Association of Protein Phosphatase PPM1G With Alcohol Use Disorder and Brain Activity During Behavioral Control in a Genome-Wide Methylation Analysis. The American journal of psychiatry 172, 543-552, doi:10.1176/appi.ajp.2014.14030382 (2015).

94 Schilling, C. et al. Cortical thickness of superior frontal cortex predicts impulsiveness and perceptual reasoning in adolescence. Molecular psychiatry 18, 624-630, doi:10.1038/mp.2012.56 (2013).

95 Schilling, C. et al. Common structural correlates of trait impulsiveness and perceptual reasoning in adolescence. Human brain mapping 34, 374-383, doi:10.1002/hbm.21446 (2013).

96 Schneider, S. et al. Maternal interpersonal affiliation is associated with adolescents' brain structure and reward processing. Translational psychiatry 2, e182, doi:10.1038/tp.2012.113 (2012).

97 Schneider, S. et al. Boys do it the right way: sex-dependent amygdala lateralization during face processing in adolescents. NeuroImage 56, 1847-1853, doi:10.1016/j.neuroimage.2011.02.019 (2011).

98 Schneider, S. et al. Risk taking and the adolescent reward system: a potential common link to substance abuse. The American journal of psychiatry 169, 39-46, doi:10.1176/appi.ajp.2011.11030489 (2012).

99 Schumann, G. Okey Lecture 2006: identifying the neurobiological mechanisms of addictive behaviour. Addiction (Abingdon, England) 102, 1689-1695, doi:10.1111/j.1360-0443.2007.01942.x (2007).

100 Schumann, G. et al. The IMAGEN study: reinforcement-related behaviour in normal brain function and psychopathology. Molecular psychiatry 15, 1128-1139, doi:10.1038/mp.2010.4 (2010).

101 Seo, S. et al. Risk profiles for heavy drinking in adolescence: differential effects of gender. Addiction biology 24, 787-801, doi:10.1111/adb.12636 (2019).

102 Shin, J. et al. Layered genetic control of DNA methylation and gene expression: a locus of multiple sclerosis in healthy individuals. Human molecular genetics 24, 5733-5745, doi:10.1093/hmg/ddv294 (2015).

103 Spanagel, R. et al. A systems medicine research approach for studying alcohol addiction. Addiction biology 18, 883-896, doi:10.1111/adb.12109 (2013).

104 Spechler, P. A. et al. The initiation of cannabis use in adolescence is predicted by sex-specific psychosocial and neurobiological features. The European journal of neuroscience 50, 2346-2356, doi:10.1111/ejn.13989 (2019).

105 Spechler, P. A. et al. Neuroimaging Evidence for Right Orbitofrontal Cortex Differences in Adolescents With Emotional and Behavioral Dysregulation. Journal of the American Academy of Child and Adolescent Psychiatry 58, 1092-1103, doi:10.1016/j.jaac.2019.01.021 (2019).

106 Spechler, P. A. et al. Cannabis use in early adolescence: Evidence of amygdala hypersensitivity to signals of threat. Developmental cognitive neuroscience 16, 63-70, doi:10.1016/j.dcn.2015.08.007 (2015).

107 Stacey, D. et al. RASGRF2 regulates alcohol-induced reinforcement by influencing mesolimbic dopamine neuron activity and dopamine release. Proceedings of the National Academy of Sciences of the United States of America 109, 21128-21133, doi:10.1073/pnas.1211844110 (2012).

108 Stacey, D. et al. A translational systems biology approach in both animals and humans identifies a functionally related module of accumbal genes involved in the regulation of reward processing and binge drinking in males. Journal of psychiatry & neuroscience : JPN 41, 192-202, doi:10.1503/jpn.150138 (2016).

109 Stein, J. L. et al. Identification of common variants associated with human hippocampal and intracranial volumes. Nature genetics 44, 552-561, doi:10.1038/ng.2250 (2012).

110 Stringaris, A. et al. Dimensions of manic symptoms in youth: psychosocial impairment and cognitive performance in the IMAGEN sample. Journal of child psychology and psychiatry, and allied disciplines 55, 1380-1389, doi:10.1111/jcpp.12255 (2014).

111 Stringaris, A. et al. The Brain's Response to Reward Anticipation and Depression in Adolescence: Dimensionality, Specificity, and Longitudinal Predictions in a Community-Based Sample. The American journal of psychiatry 172, 1215-1223, doi:10.1176/appi.ajp.2015.14101298 (2015).

112 Tahmasebi, A. M. et al. Creating probabilistic maps of the face network in the adolescent brain: a multicentre functional MRI study. Human brain mapping 33, 938-957, doi:10.1002/hbm.21261 (2012).

113 Tay, N. et al. Allele-Specific Methylation of SPDEF: A Novel Moderator of Psychosocial Stress and Substance Abuse. The American journal of psychiatry 176, 146-155, doi:10.1176/appi.ajp.2018.17121360 (2019).

114 Thompson, P. M. et al. ENIGMA and the individual: Predicting factors that affect the brain in 35 countries worldwide. NeuroImage 145, 389-408, doi:10.1016/j.neuroimage.2015.11.057 (2017).

115 Thompson, P. M. et al. The ENIGMA Consortium: large-scale collaborative analyses of neuroimaging and genetic data. Brain imaging and behavior 8, 153-182, doi:10.1007/s11682-013-9269-5 (2014).

116 Thyreau, B. et al. Very large fMRI study using the IMAGEN database: sensitivity-specificity and population effect modeling in relation to the underlying anatomy. NeuroImage 61, 295-303, doi:10.1016/j.neuroimage.2012.02.083 (2012).

117 Toro, R. et al. Genomic architecture of human neuroanatomical diversity. Molecular psychiatry 20, 1011-1016, doi:10.1038/mp.2014.99 (2015).

118 Tzschoppe, J. et al. Aversive learning in adolescents: modulation by amygdala-prefrontal and amygdala-hippocampal connectivity and neuroticism. Neuropsychopharmacology : official publication of the American College of Neuropsychopharmacology 39, 875-884, doi:10.1038/npp.2013.287 (2014).

119 Urrila, A. S. et al. Sleep habits, academic performance, and the adolescent brain structure. Scientific reports 7, 41678, doi:10.1038/srep41678 (2017).

120 Velthorst, E. et al. Genetic risk for schizophrenia and autism, social impairment and developmental pathways to psychosis. Translational psychiatry 8, 204, doi:10.1038/s41398-018-0229-0 (2018).

121 Vulser, H. et al. Subthreshold depression and regional brain volumes in young community adolescents. Journal of the American Academy of Child and Adolescent Psychiatry 54, 832-840, doi:10.1016/j.jaac.2015.07.006 (2015).

122 Vulser, H. et al. Early Variations in White Matter Microstructure and Depression Outcome in Adolescents With Subthreshold Depression. The American journal of psychiatry 175, 1255-1264, doi:10.1176/appi.ajp.2018.17070825 (2018).

123 Whelan, R. et al. Adolescent impulsivity phenotypes characterized by distinct brain networks. Nature neuroscience 15, 920-925, doi:10.1038/nn.3092 (2012).

124 Whelan, R. et al. Neuropsychosocial profiles of current and future adolescent alcohol misusers. Nature 512, 185-189, doi:10.1038/nature13402 (2014).

125 White, T. P. et al. Sex differences in COMT polymorphism effects on prefrontal inhibitory control in adolescence. Neuropsychopharmacology : official publication of the American College of Neuropsychopharmacology 39, 2560-2569, doi:10.1038/npp.2014.107 (2014).

126 Wong, C. C. & Schumann, G. Review. Genetics of addictions: strategies for addressing heterogeneity and polygenicity of substance use disorders. Philosophical transactions of the Royal Society of London. Series B, Biological sciences 363, 3213-3222, doi:10.1098/rstb.2008.0104 (2008).

127 Xu, B. et al. Impact of a Common Genetic Variation Associated With Putamen Volume on Neural Mechanisms of Attention-Deficit/Hyperactivity Disorder. Journal of the American Academy of Child and Adolescent Psychiatry 56, 436-444.e434, doi:10.1016/j.jaac.2017.02.009 (2017).

128 Yu, T. et al. Cannabis-Associated Psychotic-like Experiences Are Mediated by Developmental Changes in the Parahippocampal Gyrus. Journal of the American Academy of Child and Adolescent Psychiatry, doi:10.1016/j.jaac.2019.05.034 (2019).
